# Nature of S-States in the Oxygen-Evolving Complex Resolved by High-Energy Resolution Fluorescence Detected X-ray Absorption Spectroscopy

**DOI:** 10.1021/jacs.3c06046

**Published:** 2023-11-16

**Authors:** Maria Chrysina, Maria Drosou, Rebeca G. Castillo, Michael Reus, Frank Neese, Vera Krewald, Dimitrios A. Pantazis, Serena DeBeer

**Affiliations:** † Max-Planck-Institut für Chemische Energiekonversion, Stiftstr. 34-36, Mülheim an der Ruhr 45470, Germany; ‡ Institute of Nanoscience & Nanotechnology, NCSR “Demokritos”, Athens 15310, Greece; § Max-Planck-Institut für Kohlenforschung, Kaiser-Wilhelm-Platz 1, Mülheim an der Ruhr 45470, Germany; ∥ Laboratory of Ultrafast Spectroscopy (LSU) and Lausanne Centre for Ultrafast Science, École Polytechnique Fédérale de Lausanne (EPFL), Lausanne CH-1015, Switzerland; ⊥ Department of Chemistry, Technical University of Darmstadt, Peter-Grünberg-Str. 4, Darmstadt 64287, Germany

## Abstract

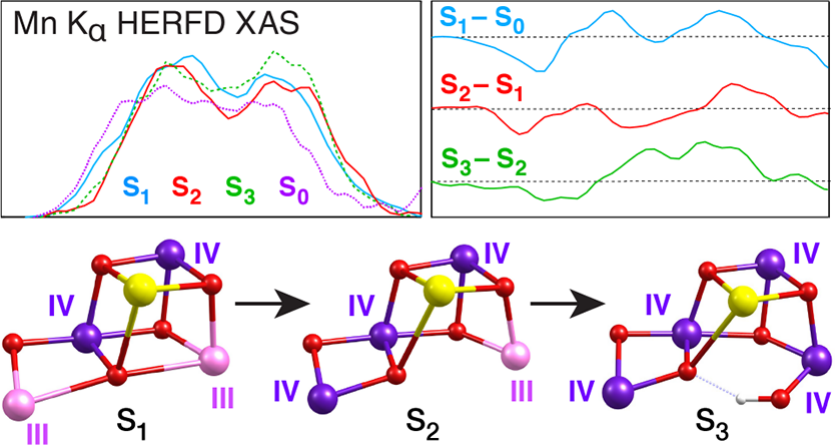

Photosystem II, the water splitting enzyme of photosynthesis, utilizes the energy of sunlight to drive the four-electron oxidation of water to dioxygen at the oxygen-evolving complex (OEC). The OEC harbors a Mn_4_CaO_5_ cluster that cycles through five oxidation states S_*i*_ (*i* = 0–4). The S_3_ state is the last metastable state before the O_2_ evolution. Its electronic structure and nature of the S_2_ → S_3_ transition are key topics of persisting controversy. Most spectroscopic studies suggest that the S_3_ state consists of four Mn(IV) ions, compared to the Mn(III)Mn(IV)_3_ of the S_2_ state. However, recent crystallographic data have received conflicting interpretations, suggesting either metal- or ligand-based oxidation, the latter leading to an oxyl radical or a peroxo moiety in the S_3_ state. Herein, we utilize high-energy resolution fluorescence detected (HERFD) X-ray absorption spectroscopy to obtain a highly resolved description of the Mn K pre-edge region for all S-states, paying special attention to use chemically unperturbed S_3_ state samples. In combination with quantum chemical calculations, we achieve assignment of specific spectroscopic features to geometric and electronic structures for all S-states. These data are used to confidently discriminate between the various suggestions concerning the electronic structure and the nature of oxidation events in all observable catalytic intermediates of the OEC. Our results do not support the presence of either peroxo or oxyl in the active configuration of the S_3_ state. This establishes Mn-centered storage of oxidative equivalents in all observable catalytic transitions and constrains the onset of the O–O bond formation until after the final light-driven oxidation event.

## Introduction

**Figure 1:**
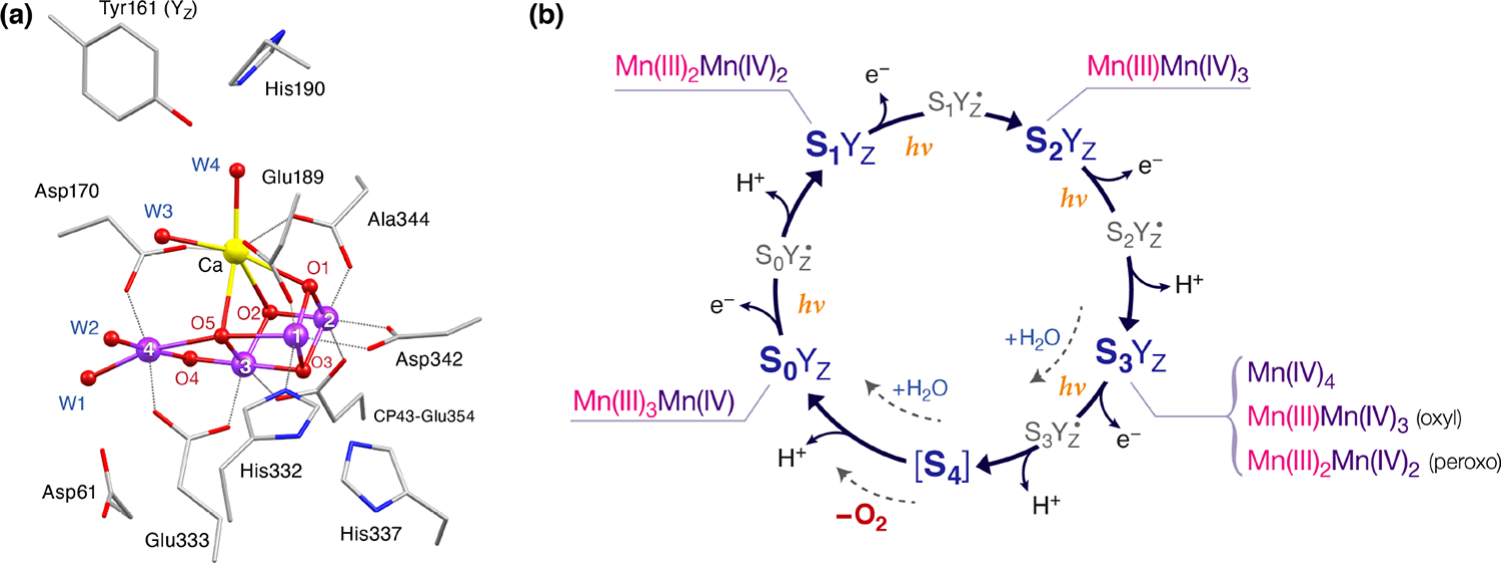
(a) Crystallographic structure of the OEC in the S_1_ state.^35^ (b) Catalytic cycle of water oxidation by the OEC with commonly accepted oxidation states of the Mn ions in the S_0_–S_2_ states and the currently discussed alternatives for the S_3_ state.

Photosystem II (PSII) of plants, algae, and cyanobacteria converts sunlight into chemical energy in the form of “high energy” electrons derived from water. These electrons are used in CO_2_ reduction to glucose, which is the primary form of food for all living organisms. PSII is thus a key enzyme in the flow of energy into the biosphere, and its unique ability to oxidize water makes its study of paramount importance both for basic research and as the only model system for developing artificial water-splitting catalysts. Water splitting takes place at the Mn_4_CaO_5_ cluster of the oxygen-evolving complex (OEC) (Figure 1a), embedded in the protein scaffold of PSII.^1−5^

The cluster cycles through a progression of four light-induced sequential oxidations, S_0_ → S_1_ → S_2_ → S_3_ → [S_4_], mediated by redox active tyrosine residue Tyr161 (Y_Z_). Dioxygen is formed and released during the last S_3_ → [S_4_] → S_0_ transition, where S_4_ is a transient state (Figure 1b). The catalytic cycle includes two water binding events, probably during or after the S_2_ → S_3_ transition and prior to reconstitution of the S_0_ state, but the details of water binding as well as the position and protonation states of the substrates for dioxygen formation remain uncertain. Commonly accepted formal oxidation states of the four Mn ions in the S_0_, S_1_, and S_2_ states are Mn(III)_3_Mn(IV), Mn(III)_2_Mn(IV)_2_, and Mn(III)Mn(IV)_3_, respectively.^6−11^ Therefore, the S_0_–S_2_ oxidation events are unanimously considered to be Mn-based. However, the localization of the S_2_ → S_3_ oxidation remains a fundamental mechanistic question since differing suggestions exist about the nature of the transition as well as the electronic structure of the S_3_-state itself.^10,12−18^

**Figure 2:**
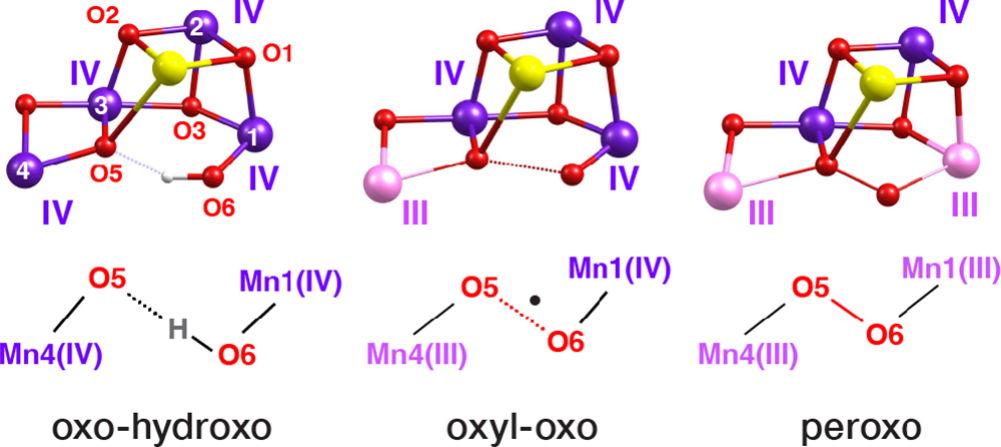
Different models discussed for the S_3_ state, showing the range of possible electronic structures.

All interpretations of electron paramagnetic resonance (EPR) observations for all different signals associated with the S_3_ state are consistent with Mn-centered oxidation in the S_2_ → S_3_ transition, leading to a Mn(IV)_4_ assignment for the S_3_ state.^19−25^ However, in X-ray absorption near edge spectroscopy (XANES) experiments, the shift of the Mn K-edge to higher energy in the S_2_ → S_3_ transition was found to be smaller than that in the S_0_ → S_1_ and S_1_ → S_2_ transitions. This observation along with K_β_ X-ray emission spectra during the S_2_ → S_3_ transition^9,15,26^ has received two alternative interpretations: either ligand-based oxidation during this transition,^15^ or a coordination sphere change from a five-coordinate Mn(III) ion to an octahedral Mn(IV) ion^10,17^ as a result of water binding (Figure 2, oxo-hydroxo). The first scenario would mean that the Mn oxidation states in the S_3_ state remain Mn(III)Mn(IV)_3_ and that a ligand radical species is formed instead (Figure 2, oxyl-oxo). This scenario appears consistent with two-flash (2F) structural models derived by recent serial femtosecond X-ray free electron laser crystallography (SFX-XFEL),^27−30^ Crystallographic data have received conflicting interpretations, ranging from an all-Mn(IV) oxo-hydroxo,^29−31^ to a ligand oxidized oxyl-oxo or peroxo species (Figure 2).^27,28,32−34^

The above distinct formulations for the S_3_ state have profound implications for the water oxidation mechanism. The possibility of oxygen-radical formation after the second flash (2F) led to the hypothesis of an “early-onset” O–O bond formation already from the S_3_ state.^18,32,36−40^ By contrast, if the S_3_ state is Mn(IV)_4_, the O–O bond formation must take place after the last flash of the cycle (3F), either in a transient S_4_ state or after formation of the S_3_Y_Z_^•^ intermediate. Computationally, all three types of models have been proposed as part of possible water oxidation mechanisms.^32,39,41,99^ However, the relative energies between computational models of the different S_3_ variants and the correspondence to the spectroscopic observations remain inconclusive.

X-ray spectroscopy provides information on core–valence transitions and can probe Mn oxidation states and the ligand environment. In previous XAS studies,^15,17^ conclusions were derived only from the XANES edge region, based on comparison with experimental results from Mn model complexes with known structures and oxidation states. The pre-edge region corresponds to the electric dipole forbidden 1s → 3d transitions, which gain intensity from metal d–p orbital mixing when the metal ion symmetry deviates from centrosymmetry, making this region sensitive to the ligand field. Correlation of the pre-edge transition energies and intensities with the structure of investigated compounds can be performed using quantum chemistry (QM) calculations.^42^ However, the resolution of partial fluorescence XAS is limited by the 1s core-hole (created upon Mn 1s excitation in XANES) lifetime broadening, and higher resolution data are required for rigorous and quantitative comparison with calculated spectra.

Herein, Mn *K*_α_ high-energy resolution fluorescence detected (HERFD) spectra of the S_0_–S_3_ states are presented. The data provide for the first time a highly resolved picture of the pre-edge region on all S-states. Importantly, contrary to previous studies,^15,17,43^ we use glycerol-free samples to avoid the glycerol-induced nonphysiological nonphysiological heterogeneity in the S_3_ state.^21,44,45^ By employing time-dependent density functional theory (TDDFT) to compute XAS spectra of a wide variety of OEC models, we achieve a detailed mapping between structural and spectroscopic features. Among other important conclusions regarding the chemical properties of distinct intermediates, correlation of the Mn XAS pre-edge region between experiment and theory shows that the HERFD spectra are consistent only with all-Mn(IV) oxo-hydroxo models of the S_3_ state, consistent with magnetic resonance studies. This result rules out ligand-based oxidation during the S_2_ → S_3_ transition and locates the onset of the formation of the O–O bond past the final light-driven oxidation in the catalytic cycle.

## Methodology

### Photosystem II Purification and Preparation of EPR-XAS Samples

Photosystem II was purified from the thermophilic cyanobacterium *Thermosynechococcus vestitus* (previously named *Thermosynechococcus elongatus*) according to Kuhl et al.^46^ Based on the final purification step, highly active PSII dimers from several preparations were pooled to reach a homogeneous sample base. The biochemical quality of the PSII dimers is routinely verified by Native PAGE electrophoresis, and oxygen evolution measurements are taken. The typical oxygen evolution activity is 5000 (±500) μmol O_2_/h · mg Chl at +30 °C. Further verification of active PSII is obtained via S-state signal progression in the EPR data. For the present batch of samples, the standard purification protocols were followed and verified by Native PAGE and EPR, but explicit O_2_ activity assays were not made for the present preparation. Given the identical EPR, however, which evidences S state advancement, a similar activity can be assumed. The final buffer contains 500 mM mannitol, 40 mM MES (pH = 6.5), 10 mM CaCl_2_, 10 mM MgCl_2_, and 0.03% v/v *n*-dodecyl β-D-maltoside. Phenyl-para-benzoquinone (PPBQ) dissolved in dimethyl sulfoxide (DMSO) was added as electron acceptor at a final concentration of 0.5 mM. Photosystem II (1.5 mM Mn, 15 μL) was loaded in XAS cells. The cells were designed to fit in the EPR resonator and the cryostat utilized for HERFD XAS measurements. Both surfaces of the cell were open and sealed with Mylar tape windows. The sample dimensions in the cell were 2.5 mm width and 8 mm length and 0.8 mm depth. Samples were synchronized in the S_1_ state by a “pre-flash” and dark incubation at room temperature for 1 h. All samples were illuminated by a Nd:YAG laser (wavelength 532 nm, 10 ns pulse of 300 mJ) with 0, 1, 2, or 3 flashes and frozen in liquid N_2_. The laser beam was split in two and the sample was illuminated from both sides.

### EPR Quantification of S-States

The S-state advancement is imperfect.^47−55^ By using short intense laser pulses, we can avoid “double hits”, but it is impossible to avoid “misses”. The miss factor was calculated using the intensity of the multiline EPR signal of S_2_ state in all samples (Figure S1), as previously reported by Messinger et al.^15^ Measurements were carried out by using a Bruker E500 spectrometer equipped with a Bruker ER 4116DM resonator and an Oxford Instruments ESR 935 cryostat. From the peak to peak intensity of the multiline (lines used marked in Figure S1a), the population of each S-state after 0, 1, 2, and 3 flashes was calculated (Tables S1, S2) and used in order to deconvolute the pure S_1_, S_2_, S_3_, and S_0_ XAS spectra. We note that after the fourth flash the intensity of the S_2_ multiline signal is 80% compared to the first cycle (Figure S1b). The very good conversion factor confirms the high activity of the samples. Given that inactive OEC centers would have released Mn(II) in solution, all samples were checked for the presence of Mn(II) EPR signals and batches with high Mn(II) content were discarded. Crucially, the homogeneity of our S_3_ state samples has been confirmed by W-band EPR spectroscopy (Figure S2).^44^

### HERFD Measurements

Mn Kα HERFD measurements were performed at beamline 6–2 of the Stanford Synchrotron Radiation Lightsource (SSRL).^56^ The SPEAR storage ring was operating at 3 GeV with a current of 500 mA. BL6–2 utilizes a 56-pole 0.9 T wiggler insertion device. Two Rh-coated Si crystals (one flat for vertical collimating and one cylindrical for focusing, upstream and downstream of the monochromator) were utilized to focus the beam. The incident beam was monochromatized using a pair of cryogenically cooled Si(311) crystals, giving an energy resolution of ∼0.2 eV. The flux was estimated to be ∼2.9 × 10^11^ photons/s in a 420 × 200 μm beam spot. The energy of the incident beam was calibrated by setting the maximum of the pre-edge peak of KMnO_4_ to 6543.21 eV. Kα-HERFD and Kα XES data were collected by utilizing a Johann-type XES spectrometer equipped with five Ge(111) crystals and an energy-dispersive silicon drift detector. The spectrometer resolution at the elastic peak was 0.8 eV. The sample temperature during measurements was poised at 10 K by using liquid He-cooled flow cryostat. The beam shape was Gaussian with vertical full width at half height of 200 μm and horizontal of 420 μm. By using a vertical step of 300 μm and horizontal step of 630 μm, it was possible to measure ∼50 separate spots per sample. The Kα HERFD scans were collected in an energy range of 6530–6570 eV, while longer 6530–6800 eV scans were measured to facilitate proper normalization. The energy step was varied across the measurement and was 0.15 eV for the pre-edge region, which is the primary focus of the present study, and 0.2 eV across the edge. The Kα XES spectra were collected to assess the Kα maxima of each S state, and hence, short scans were used in the energy range of 5896–5904 eV.

### Damage Studies

Detailed damage studies were performed before the actual measurement in all S states (Figure S3). They involve measurement of XAS scans in the range 6535–6570 eV in order to establish the maximum dwell time per sample spot. Due to the low sample concentrations, we exposed to radiation a number of spots and collected consecutive scans in each spot. We compared the average of all the first scans, to the average of all the second scans and so on, to establish the time point at which a change in the pre-edge and edge region is observed. Attenuation of the incident beam by using foils was also required. A range of attenuation factors was tested, and we concluded that 6% of the total flux was not damaging the sample during a scan time of ∼60s, while reasonable signal/noise was maintained. Additionally, time scans were performed at a constant incident energy of 6551 eV, the energy point that was considered as the most sensitive probe of damage during the initial damage scans. The dwell time for S_1_ and S_2_ states was determined as ∼100 s/spot while for S_3_ and S_0_ was ∼75 s/spot and was used during the actual measurement. Higher oxidation states of the Mn_4_Ca cluster are expected to damage faster. This fact was verified by the damage studies, and a shorter scan (75 s vs 100 s/spot) was used for the S_3_-state. We note that while a shorter dwell time for the formally more reduced S_0_ may seem counterintuitive, this is due to the fact that the S_0_ samples as prepared contain a significant amount of S_3_ as quantified by EPR. A dose of 1.7 × 10^7^ photons/μm^2^ was used for the S_1_ state, 1.4 × 10^7^ photons/μm^2^ for the S_2_ state and 1.2 × 10^7^ photons/μm^2^ for the S_3_ and S_0_ states.

### HERFD Data Processing

Every scan was normalized to the incident flux *I*_0_ and subsequently scans of the same flash number were averaged in PyMCA in order to create the average of 0, 1, 2, and 3 flash spectra (ca. 150 scans were averaged for the S_1_ and S_2_, while ca. 220–230 scans for the S_3_ and S_0_ states). All other processing was performed in Matlab. The short scans (6530–6570 eV) were normalized to the edge jump by using the long scans (6530–6800 eV). Spectra of pure S_*i*_ states were acquired by subtraction of the appropriate percentage of pure S_*i*–1_ (and S_*i*–2_ if needed) as calculated by EPR quantification of S-states after each flash (Tables S1 and S2). The rising edge of the pure spectra was fitted by interpolating a spline polynomial in the region 6530–6550 eV. By subtraction of the XAS spectrum minus the fitted line, the isolated pre-edge spectra of the S-states were acquired. Fitting of the pre-edge peaks was performed using Voigt curves (Lorentzian has only a small contribution) and least-squares fitting. The fact that the peaks represent Gaussian shape is expected since the 1s core hole lifetime broadening (Lorentzian) is effectively eliminated in a HERFD experiment, and the energy resolution is thus dominated by the instrumental broadening (Gaussian).

### Evaluation of the Error

The error of the 0, 1, 2, and 3 flash spectra was estimated by the standard error of the mean of averaged spectra (Figure S4). This is a measure of how far our sample mean, i.e., scans of our experiment, is likely to be from the true population mean that would be infinite scans that would give the real spectrum. The standard error is calculated as  where  is the standard deviation, *x̅* is the mean of scans *x*_1_–*x*_*n*_, and *n* is the total number of scans. After subtraction of the appropriate percentage of the S_*i–*1_ state determined by EPR from the raw spectra in order to acquire the pure S_*i*_ state spectra and renormalization, the error was recalculated as , where  is the standard error of the pure spectrum of the S_*i*_ state, *X*_*S*_i__ is the fraction of the S_*i*_ state in the sample, while *X*_*S*_*i*–1__ is the fraction of the “missed” centers in the S_*i–*1_ state that was subtracted, σ_*S*_i__ is the standard error of the spectrum of S_*i*_, and σ_*S*_*i*–1__ is the standard error of the S_*i–*1_ spectrum.

### Computational Details

Cluster models of the OEC were constructed from the X-ray crystallographic coordinates of the 5B66 structure monomer B.^35^ The models include the inorganic core Mn_4_CaO_5_, terminal water molecules W1–W4, and first coordination sphere amino acids His332, Glu189, Asp342, Ala344, CP43-Glu354, Asp170, and Glu333. Second coordination sphere residues include His337, CP43-Arg357, Asp61, Ser169, Leu343, Tyr161, His190, Asn298, Gln165, Val185, and Phe186, and 13 crystallographic water molecules: 6 from the O1 water channel, 3 hydrogen bonding to W3 of the Ca^2+^ ion, 2 from the Cl^–^ water channel, and 2 from the O4 water channel. Overall, the S_1_ state model (with W2 = H_2_O) includes 329 atoms and is shown in Figure S5.

All calculations were performed with Orca 5.^57^ Geometry optimizations were carried out using the B3LYP^58,59^ functional with the resolution of the identity (RI) approximation including D4 dispersion corrections.^60^ Optimizations were performed in the respective broken symmetry states for each model, i.e. α–β–α–β for **S**_**1**_**A**, **S**_**1**_**HA**, and **S**_**0**_**O5**, β–α-α–α for **S**_**1**_**B**, **S**_**1**_**HB**, **S**_**3**_**P**, and **S**_**0**_**HO4**, α–β–β–α for **S**_**1**_**O5H**, **S**_**2**_**A**, **S**_**2**_**HA**, and **S**_**0**_**O4**, α–α–α–β for **S**_**2**_**B**, **S**_**2**_**HB**, **S**_**2**_**O4H**, **S**_**3**_**AW**, and **S**_**3**_**BW**, α–β–α–α for **S**_**0**_**HO4**, and α–α–α-α–β (O5–O6 radical antiferromagnetically coupled to Mn ions) for **S**_**3**_**O**. Relativistic effects were considered throughout using the zeroth-order regular approximation (ZORA).^61−63^ The scalar-relativistically recontracted^64^ ZORA-def2-TZVP(-f) basis sets^65^ were used for all atoms except C and H for which the ZORA-def2-SVP basis sets were used. In all calculations, the CPCM solvation^66^ with dielectric constant ε = 6.0 was used to simulate the effect of the protein surrounding.

XAS spectra were calculated with the TD-DFT method employing the Tamm–Dancoff approximation.^67,68^ The TPSSh functional^69^ was used and the computed excitation energies were shifted to higher energies by 36.3 eV, to account for systematic methodological deviations, based on previous benchmarking studies on Mn monomers and dimers.^70,71^ The accuracy of the method has been quantified in previous benchmark studies on mononuclear Mn complexes using the computed *R*^2^ of the linear relationship between the calculated and experimentally determined transition energies (*R*^2^ = 0.94) and intensities (*R*^2^ = 0.88).^71^ The RI and chain of spheres (RIJCOSX) approximations^72^ were employed to speed up the calculations. The XAS spectra of each Mn ion were calculated with 150 roots, and the spectra of each model were plotted by adding the spectra of the four Mn ions and using a peak Gaussian broadening of 1.1 eV. In order to compare with the normalized intensities of the experimental spectra, all computed intensities were multiplied by 0.02, based on agreement between the maximum intensity of the 0F state with the computed intensities of the S_1_ states.

## Results and Discussion

### HERFD Experiment

**Figure 3:**
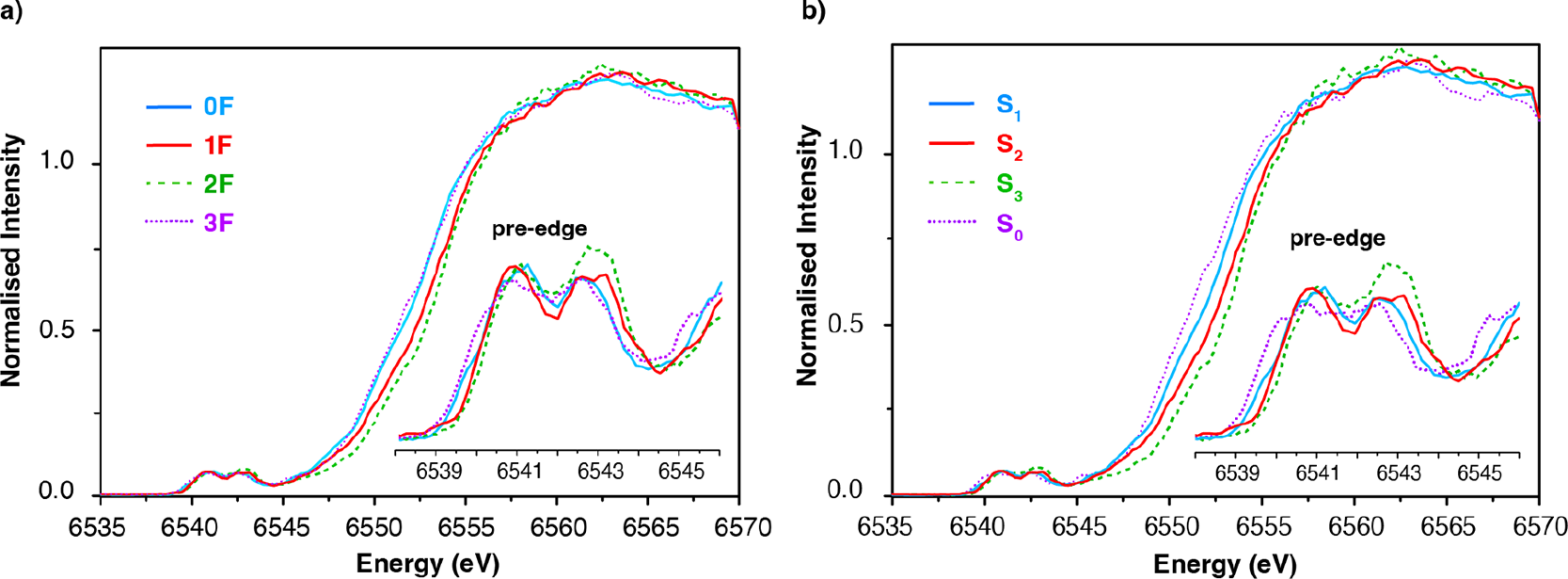
(a) HERFD spectra of the dark-adapted samples (0F) and after 1–3 flashes (1–3F). Spectra are normalized to the edge jump. 3-point adjacent averaging was used. (b) Pure spectra of all S-states after the appropriate subtractions based on EPR quantification.

The Mn Kα HERFD X-ray absorption spectra of the dark-adapted S_1_ state, the one-flash (S_2_), two-flash (S_3_), and three-flash (S_0_) illuminated states of PSII samples from *T. vestitus* were measured at beamline 6–2 of the Stanford Synchrotron Radiation Lightsource (SSRL) (Methods section, SI) and are shown in Figure 3a. All spectra are normalized to the edge jump, as described in the Methods. Functionality of the samples is indicated by the period four oscillation in the S_2_ state multiline *g* ≈ 2 EPR signal (Figure S1).

Progression of PSII samples to the next S-state upon illumination is not perfect; thus, quantification of the population of PS II centers of the sample in each S-state is essential. EPR spectroscopy was employed following a previously suggested protocol.^15^ Quantification of the flash-induced S-state progression efficiency was performed based on the intensity of the multiline *g* ∼ 2 EPR signal of the S_2_ state after each flash.^15^ The S_2_ population after each visible light flash was quantified, and a model considering misses for every flash illumination was fitted to the EPR data. The miss factor was calculated to be 6%. In other studies, miss factors of 6–18% have been reported.^10,15,17,19,43^ In a separate experiment, a singly flashed sample was illuminated continuously at 200 K in order to obtain the maximum of S_2_ and no increase in the multiline signal intensity was observed. Double hits did not improve the fitting. A considerable population of PS II centers (15%) remains in the S_2_ state and does not progress to S_3_ and S_0_ upon illumination. This is caused by acceptor side deficiency: the acceptor side in some PSII centers cannot accept more electrons in order for the OEC to proceed further than S_2_. Single turnover values between ∼5 and 10% of PSII centers have been reported elsewhere.^10,15^ A percentage (∼5%) of centers in S_2_ state in the dark adapted sample has also been considered, but the inclusion of such population did not improve the fitting (see also SI, section 1 and Table S1). The population of each S-state after each laser pulse is presented in Table 1. After “0, 1, 2, 3 flashes”, there is 100% S_1_, 94% S_2_, 74% S_3_, and 70% S_0_, respectively.

**Table 1:** S-State Content (%) after a Given Number of Flashes

flash	S_1_	S_2_	S_3_	S_0_
0	**100**			
1	6	**94**		
2	1	25	**74**	
3	0	16	14	**70**

The optimal fitting with the experimental data was achieved by a miss factor of 6% and assuming that a 15% population of the OEC centers remains blocked in the S_2_ state. The pure S-state spectra derived after deconvolution according to the S-state quantification are shown in Figure 3b. The spectra consist of the rising edge at 6545–6560 eV, that represent 1s → 4p transitions, and the pre-edge at 6538–6545 eV, attributed to dipole forbidden 1s → 3d transitions. Each of the regions is examined separately in the next sections.

### Analysis of the Edge Region

For systems with similar coordination environment, the energy of the edge is dependent on the oxidation state of the metal ion: as the oxidation state increases the edge shifts toward higher energy. Visual inspection of the edge regions in Figure 3b shows that the edge shifts to higher energies upon the S_0_ → S_1_ and S_1_ → S_2_ transitions. For the S_1_ → S_2_ transition, the edge shift can be attributed only to the more positive charge of the cluster since the structures of S_1_ and S_2_ are similar, as proposed by EXAFS^17,43^ and most recently by the XFEL crystal structures.^29^ The S_0_ → S_1_ transition involves deprotonation in addition to oxidation; therefore, the observed edge shift is attributed to Mn-centered oxidation as well as small structural rearrangements. Thus, in agreement with previous works,^15,17,43^ positive edge energy shifts are observed during the S_0_ → S_1_ and S_1_ → S_2_ transitions, which reflect Mn-based oxidations.

The edge shift during the S_2_ → S_3_ transition is not equally obvious from visual inspection because the shapes of the edge of S_2_ and S_3_ states are significantly different, as shown also in the first derivatives of the spectra (Figure S6). Those differences possibly stem from significant structural changes involved in the S_2_ → S_3_ transition, which include deprotonation and possibly the insertion of a water molecule in the cluster. Thus, in S_2_ → S_3_, the edge shift is dependent on the oxidation state changes as well as the ligand field change; since the magnitudes of these effects are comparable, this complicates the interpretation of the edge as it has been shown in Mn model systems.^73^ A change in the coordination environment of a Mn ion from 5-coordinate Mn(III) to 6-coordinate Mn(IV) was proposed based on comparison of Kβ emission difference spectra of the S_3_ minus the S_2_ state with the difference spectra of synthetic models with Mn(III)L_5_, Mn(IV)L_5_, and Mn(IV)L_6_.^9^

Three different methods were used for assessing the energy of the edge in XAS experiments: (a) the position at the half intensity of the normalized spectra,^74^ (b) the integral method,^75^ and (c) the energy at the inflection point.^15^ In Table 2, the energies of the edge and the edge shifts determined using all of the above methods are given. The half intensity energy point, after proper normalization using the long scans up to 6800 eV, shifts ∼0.7 eV with each S-state transition from S_0_ to S_3_. Similar results are obtained with the integral method, which gives a shift of ∼0.6 eV. By contrast, the inflection point of the edge, determined by the second derivative (Figure S7), is almost the same for the S_2_ and S_3_ states. Notably, the present results for the inflection point values are similar to those reported by Messinger et al.,^15^ while those reported with the integral method are consistent with Haumann et al.^17^ As shown in Figures S8 and S9, the present XANES spectra are very similar in the rising edge region to the previously reported XANES measured on spinach^15,17^ and cyanobacteria.^43^ The inflection point method is more sensitive to the shape of the edge since it is defined by a single point. Thus, the shift of the inflection point energy is not consistent with the other two methods and the different methods used by the different groups for assessing the edge shift may cause the discrepancy in the literature regarding the presence^17,74,76^ or near absence^15,77^ of edge shift during S_2_ → S_3_ and thus if Mn-centered or ligand-based oxidation occurs during this transition.^12,22,32,38,41,78−81^

**Table 2:** Edge Energies and Edge Shifts (S_*i*_–S_*i*-1_, in Parentheses) Obtained with Three Different Methods^a^

	half of normalized Intensity	integral method	inflection point
S_0_	6550.8	6551.6	6550.5
S_1_	6551.4 (0.6)	6552.1 (0.5)	6553.1 (2.6)
S_2_	6552.2 (0.8)	6552.7 (0.6)	6553.7 (0.6)
S_3_	6552.9 (0.7)	6553.2 (0.5)	6553.9 (0.2)

a All energies in eV.

Overall, all methods of determining the edge energy confirm Mn-based oxidation in the S_0_ → S_1_ and S_1_ → S_2_ transitions. On the other hand, diverging results are obtained in the case of the S_2_ → S_3_ transition by different methods, which has given rise to seemingly contradictory interpretations among different groups, despite their data being almost identical. Given the additional complication of structural differences between the S_2_ and S_3_ states, the locus of oxidation in this transition cannot be conclusively determined from the analysis of the edge region. Therefore, in the next sections we focus on the analysis of the pre-edge region, which more directly correlates with the electronic structure, and where the high resolution of the HERFD spectra combined with QM calculations provide an unambiguous interpretation.

### Analysis of the Pre-edge Region

**Figure 4:**
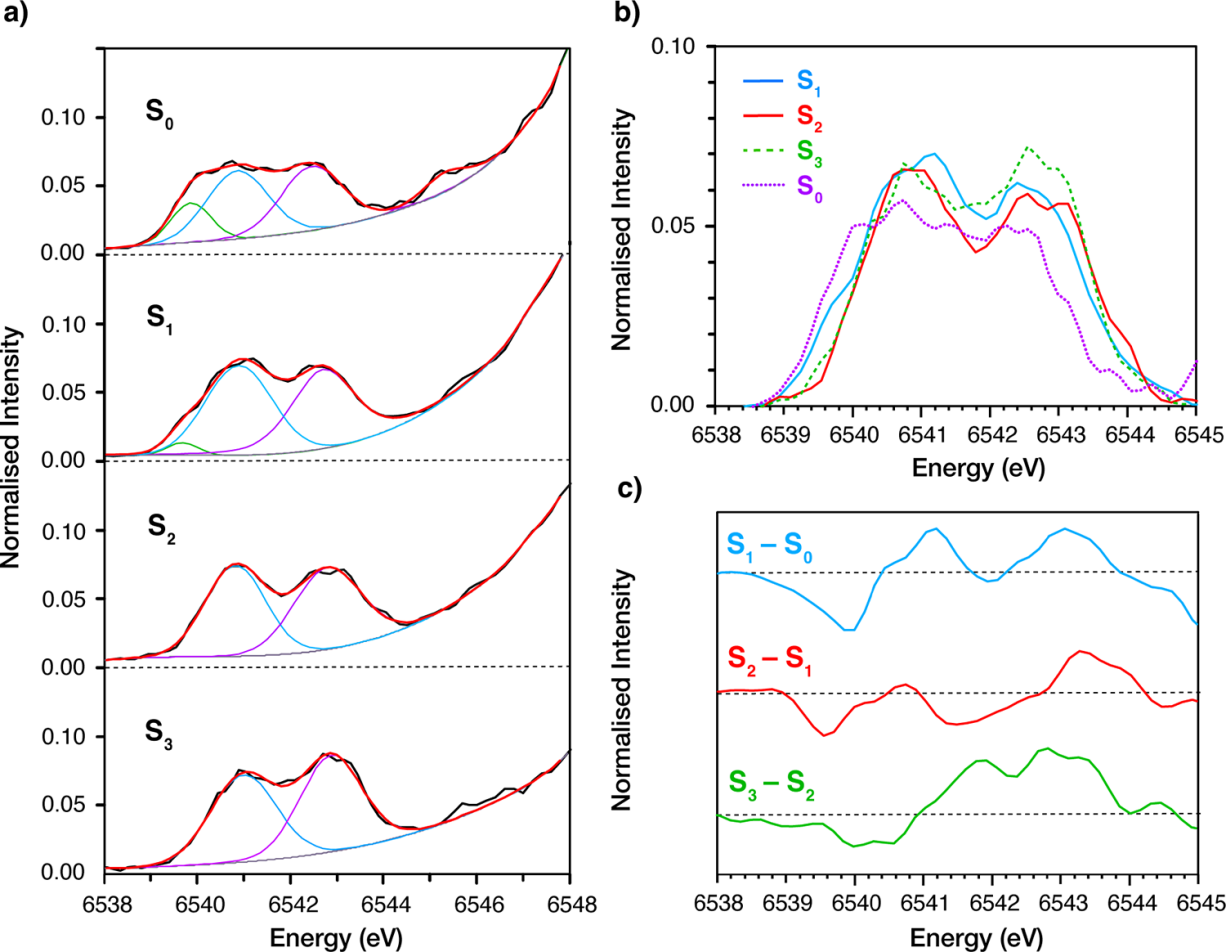
(a) Fitting of the pre-edge region with Gaussian peaks and the rising edge background. (b) Pre-edge region of each S-state after the subtraction of the rising edge background. (c) S-state pre-edge XAS spectral differences. The dashed lines show the zero value for each difference spectrum.

Figure 4 shows the expanded pre-edge region for all S-states. Before analyzing the current data in more detail, it is important to compare these spectra to previous XAS data. As discussed above, the XANES region of the HERFD spectra are essentially identical to previous reports.^15,17,43^ However, as seen in Figure S8, the ratio of intensities in the pre-edge region is modulated relative to previous reports. First, we note that the present data represent the highest resolution XANES data available for all S states. While 1s2p RIXS planes of the S-states were previously reported by Glatzel et al.,^82^ these data do not include the full XANES and thus do not allow for proper normalization. Further, the previous data were collected with a Si(111) monochromator relative to the Si(311) monochromator used in the current study. Hence, we estimate the previous report would have a resolution of ∼1.3 eV at constant emission energy relative to the ∼0.8 eV resolution in the current study. These differences in resolution, however, are unlikely to account for the differences in the pre-edge ratios. More likely, the observed differences are attributed to the fact that no glycerol was used in the present study. As glycerol is known to change the equilibrium of the different configurations of the S states, we hypothesize that this may be the origin of the modulations in the pre-edges. This is consistent with previous studies by Yano et al.,^45^ which showed that near-infrared conversion of the S_2_ low spin multiline signal to a high-spin EPR signal is correlated with a decrease in the intensity of the first pre-edge feature. These observations thus further motivate the importance of having the present data set for all S-states in the absence of glycerol. In order to quantitatively correlate the present data with theory, we proceeded to a detailed fitting analysis.

The pre-edge region of each state was fitted by Gaussian curves, and the optimal fittings are shown in Figure 4a. The pre-edge regions of the S_0_ and S_1_ states can be best described by three features: a lower intensity peak at 6540 eV and two higher intensity peaks at 6541 and 6543 eV. The S_2_ and S_3_ pre-edge were fitted by only two peaks at 6541 and 6543 eV. Alternative fittings are given in Figure S10 and Table S3.

Changes in the pre-edge region with S-state progression can be quantified based on the areas of the individual peaks and of the overall pre-edge region, given in Table 3. The intensity of the first peak decreases from the S_0_ to the S_1_ peak, and the peak is not detectable at all at S_2_ and S_3_. The second peak at 6541 eV becomes more intense in S_1_ than in S_0_ and has a similar intensity in S_1_–S_3_. A subtle peak is also observed at ∼6546 eV in the S_0_ spectrum partially covered by the rising edge. The third peak at 6543 eV increases from S_0_ to S_1_ and from S_2_ to S_3_, but not in S_1_ to S_2_. In order to compare solely the contributions to the XANES spectra due to the 1s → 3d transitions, the contribution of the rising edge to the pre-edge was subtracted. The spectra of S_0_–S_3_ with the subtracted baseline are shown in Figure 4b. The pre-edge region changes with S-state progression are shown in the difference spectra in Figure 4c. Figure S11 indicates the standard errors of the difference spectra.

**Table 3:** Energy in eV and Area (in parentheses) for Each Fitted Peak in Each S-State Pre-edge Spectrum and Intensity Weighted Average Energy (IWAE) and Area of the Total Pre-edge Region

	peak 1	peak 2	peak 3	total
S_0_	6539.9(0.031)	6540.9(0.082)	6542.5(0.079)	6541.4(0.208)
S_1_	6539.7(0.008)	6540.9(0.118)	6542.7(0.115)	6541.6(0.235)
S_2_		6540.8(0.108)	6542.8(0.108)	6541.8(0.217)
S_3_		6541.0(0.113)	6542.9(0.121)	6542.0(0.238)

The changes in the overall shape of the pre-edge region during the S_0_–S_3_ state transitions can be expressed through the intensity weighted average energies (IWAEs). The IWEAs shift 0.2 eV higher after each transition, which imply that higher energy transitions gain intensity with each Mn oxidation. Notably, those changes cannot be explained solely in terms of Mn oxidation during each transition, as in the case of the edge region. Apart from indirect correlations between pre-edge intensities and the presence of specific groups,^83^ a detailed correlation between structure and spectroscopy requires QM calculations.^70,71,83−86^ Therefore, in the next sections, we attempt to correlate the pre-edge features with structural features using QM-derived models of all states.

### Correlation with Structural Models

**Figure 5:**
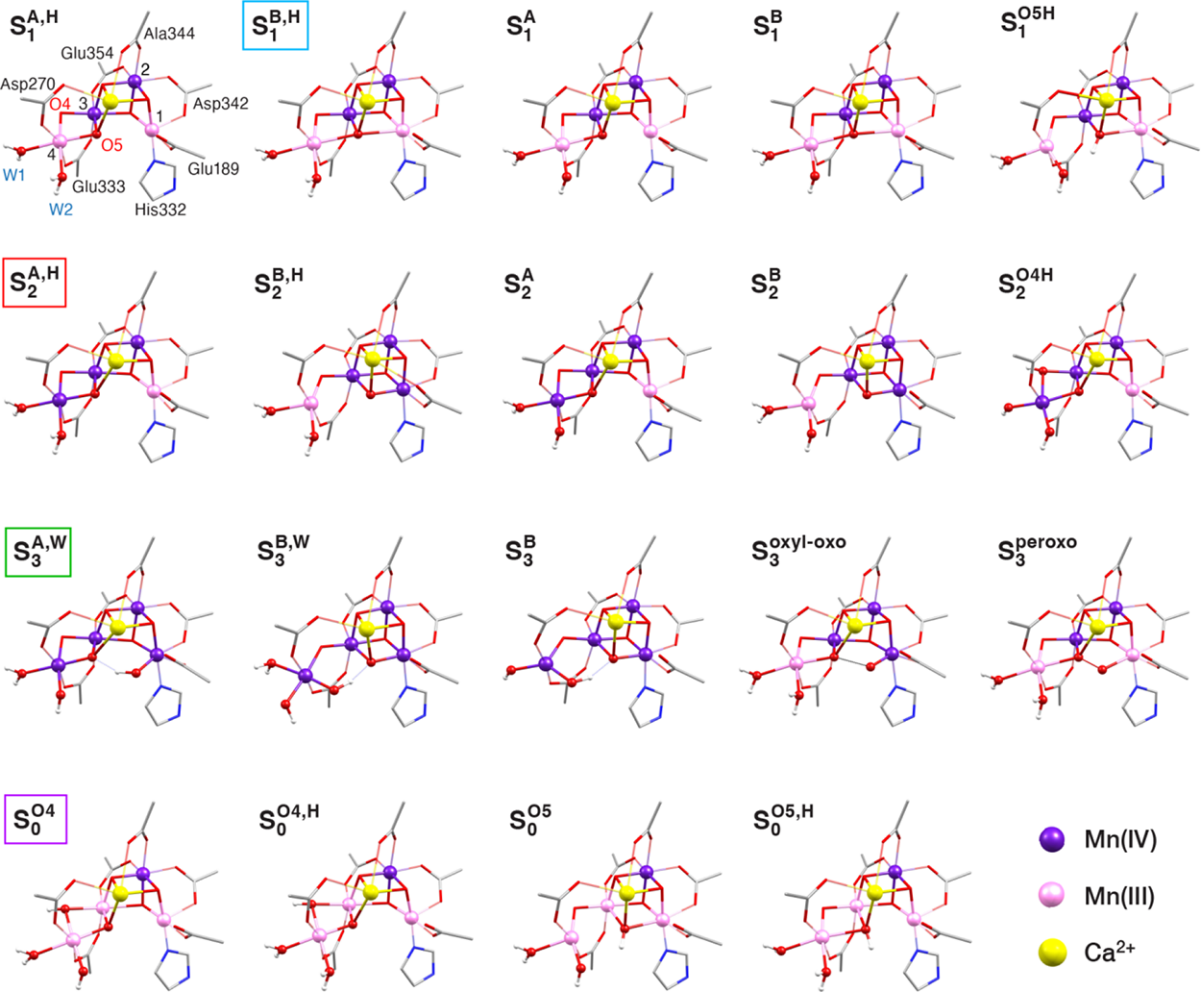
Optimized core structures of the computational models of S_0_–S_3_ states considered in this work. For clarity, only the inorganic core and a few selected coordinating ligands (of the actual ∼330 atom models) are depicted in this figure. Mn(III) ions are shown in light pink, and Mn(IV) ions are in dark purple. Label superscripts A and B are for “open” and “closed” cubane geometries, respectively; H superscript denotes that W2 is in the aquo form (H_2_O), otherwise, it is in the hydroxo form, and O4 and O5 denote that bridging oxo ligands O4 and O5, respectively, are protonated in the specific models. The models whose labels are in colored boxes turned out to be in best agreement with experiment, as explained in the following sections.

To correlate the XAS spectra with the structure of the OEC in each state, we optimized models of several variants of the S_0_–S_3_ states, and we calculated their XAS spectra. The core parts of all optimized structures are shown in Figure 5, along with explanations of the labeling used in this work. Additionally, the inorganic cores of all models are depicted schematically in Figure S12 where the Jahn–Teller axis orientations for all Mn(III) ions are depicted and important distances are indicated. For the S_1_ state, we considered five models with variations in the protonation states of terminal water-derived ligands on Mn4 and the O5 bridge, as well as in the orientation of pseudo-Jahn–Teller elongation axes of the Mn(III) ions.^87^ In all optimized S_1_ state models, the Mn oxidation states are III–IV–IV-III. For the S_2_ state, we considered five models that take into account all different ideas discussed in the literature regarding protonation states of terminal water-derived ligands and the O4 bridge,^88^ as well as valence isomerism (i.e., Mn oxidation states III–IV–IV−IV and IV–IV–IV−III).^89^ For the S_3_ state, we considered all possibilities discussed in the introduction regarding Mn and ligand oxidation states, i.e., Mn IV–IV–IV−IV with oxo and hydroxyl O5 and O6 ligands, III–IV–IV−IV with oxo-oxyl radical, or III–IV–IV−III with peroxo O–O bond formation, in total 5 models. Finally, for the S_0_ state we considered 4 models differing in the protonation states of W2 and of the O4 and O5 bridges.^90,91^ The TD-DFT calculated XAS spectra of all 19 optimized structures are shown in Figure S13, compared to the experimental spectra for each S-state.

### Analysis of Difference Spectra

The difference spectra between successive S-states are substantially more informative compared to the individual spectra, as it removes systematic errors in the intensity evaluation and explicitly isolates the features that change from one state to the next. The results show that in some cases where the similarities in computed spectra are significant, the difference spectra enable the confident selection of the best fitting structural models.

**Figure 6:**
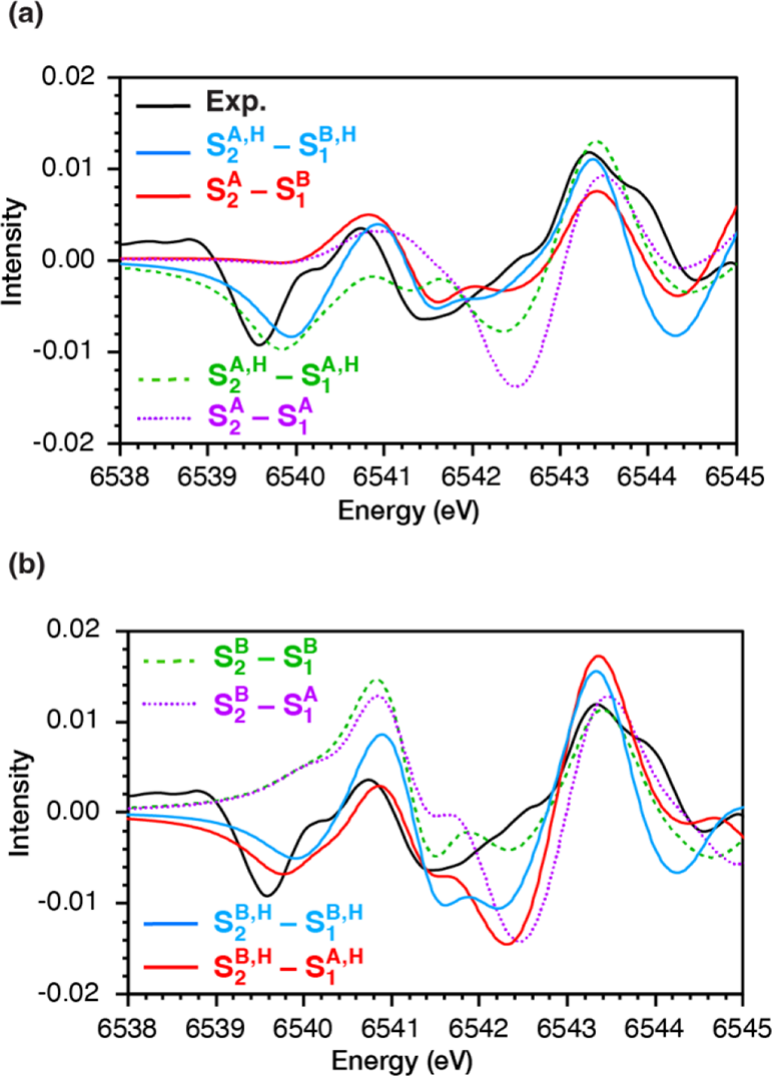
Experimental and calculated S_2_–S_1_ spectral differences for different S_1_ and S_2_ isomers.

Figure 6 compares the S_2_–S_1_ difference spectra between experiment and theory for the selected computational models. Importantly, the comparison of difference spectra using distinct computational models is so sensitive that it is possible to distinguish between S_1_ state isomers in which the orientation of the Jahn–Teller axis of the Mn4(III) ion is either perpendicular or collinear to the Mn1(III) Jahn–Teller axis (**S**_**1**_^**A,H**^ and **S**_**1**_^**B,H**^, respectively),^87^ but also between different protonation states of the terminal W2 ligand in both the S_1_ and S_2_-state models. For example, the S_2_–S_1_ difference spectra for the S_1_ models with perpendicular Jahn–Teller axes (models **S**_**1**_^**A**^ and **S**_**1**_^**A,H**^) show a large negative intensity difference at 6542–6543 eV, inconsistent with experiments, whereas this is well reproduced with model **S**_**1**_^**B,H**^. Interestingly, the observed negative intensity difference in the region 6539–6540 eV is only reproduced by S_1_ models that have W2 in the aquo form (in agreement with FTIR studies^92^), but not by those with W2 in the hydroxo form. This is associated with the shoulder at 6539.5 eV that is observed for the S_1_ models where W2 is H_2_O (Figure S13a).

The models that best reproduce the experimental S_2_–S_1_ difference spectra are **S**_**1**_^**B,H**^ and **S**_**2**_^**A,H**^ (Figure 6a, blue line). The comparison shows the excellent agreement between the experiment and calculations. It is worth pointing out that the calculated XAS spectrum of **S**_**2**_^**B,H**^ is slightly lower in intensity than **S**_**2**_^**A,H**^ (Figure S13b), similar to the S_2_ high-spin and low-spin forms reported by Chatterjee et al.^45^ By contrast, the computed spectrum of the S_2_ state model with protonated O4, (**S**_**2**_^**O4H**^), which has been suggested as a possible interpretation of the high-spin S_2_ form,^88,93^ is more intense than **S**_**2**_^**A,H**^ (Figure S13b). Therefore, our experimental pre-edge results combined with TD-DFT calculations are most consistent with valence isomerism^89^ giving rise to the low and high spin S_2_ state signals, respectively. As the samples were not treated with glycerol,^94^ about 20% of the centers are in the high-spin S_2_ state (quantification based on EPR, see SI). Since the fraction of high-spin S_2_ is small, the effect on the S_2_–S_1_ difference spectra in the pre-edge region is negligible (Figure S14) and thus we use only the dominant **S**_**2**_^**A,H**^ spectrum for the difference spectra plots.

Comparison of the calculated S_1_–S_0_ difference spectra using different computational models, in Figure S15, shows that model **S**_**0**_^**O4**^ exhibits the best fitting with experiment among the different S_0_ variants. Notably, light-induced Fourier transform infrared (FTIR) S_1_–S_0_ difference spectra reported by Yamamoto et al.^95^ also indicate O4 protonation in the S_0_ state; however, that study also supports a fully protonated W2, which would be represented by our **S**_**0**_^**O4,H**^ model. This would indeed be more consistent with one electron oxidation and one deprotonation (of O4) during the S_0_ → S_1_ transition, and it needs to be further investigated. Previous benchmarking studies on Mn oxo-bridged compounds have also demonstrated the sensitivity of Mn XAS to oxo-ligand protonation states.^70^ Therefore, analysis of the S_0_–S_2_ state transitions shows that the pre-edge region combined with quantum chemistry simulations not simply confirms Mn-based oxidation but directly constrains the possible structural features of each state.

**Figure 7:**
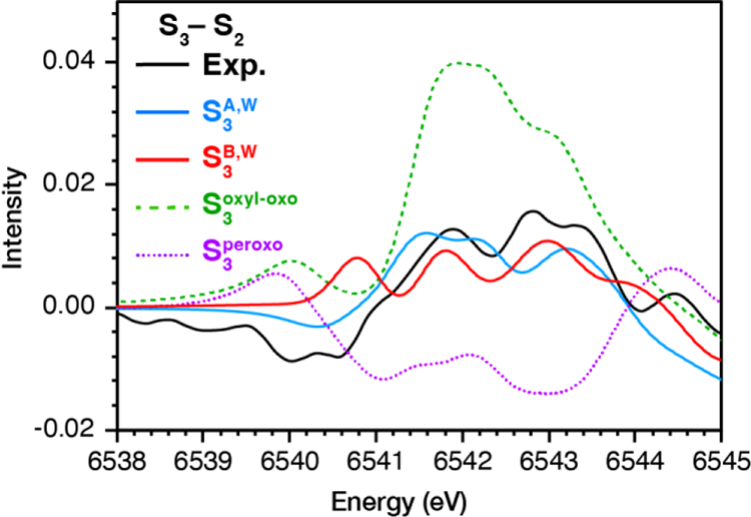
Experimental S_3_–S_2_ data and calculated S_3_–S_2_ difference spectra for models **S**_**3**_^**A**, **W**^, **S**_**3**_^**B**, **W**^**S**_**3**_^**oxyl**–**oxo**^, **S**_**3**_^**peroxo**^, and **S**_**2**_^**A**, **H**^.

Even more pronounced than the S_1_ and S_2_ states are the differences observed among computational models of the S_3_ state in the case of S_3_–S_2_ difference spectra shown in Figure 7. The comparison reveals that the Mn(IV)_4_ oxo-hydroxo models (**S**_**3**_^**A,W**^ and **S**_**3**_^**B,W**^) are clearly in better agreement with experiment than the Mn(III)Mn(IV)_3_ oxyl-oxo (**S**_**3**_^**oxyl–oxo**^) and Mn(III)_2_Mn(IV)_2_ peroxo (**S**_**3**_^**peroxo**^) models. The **S**_**3**_^**oxyl–oxo**^ and **S**_**3**_^**peroxo**^ are completely inconsistent because **S**_**3**_^**oxyl–oxo**^ shows much higher intensity than S_2_ between 6541 and 6544 eV, whereas **S**_**3**_^**peroxo**^ shows much lower intensity. Among the Mn(IV)_4_ oxo-hydroxo structural isomers, **S**_**3**_^**A,W**^ and **S**_**3**_^**B,W**^, the open cubane **S**_**3**_^**A,W**^ shows the closest agreement with experiment. The above results strongly disfavor ligand-based oxidation during the S_2_ to S_3_ transition.

### Analysis of S-State Progression

**Figure 8:**
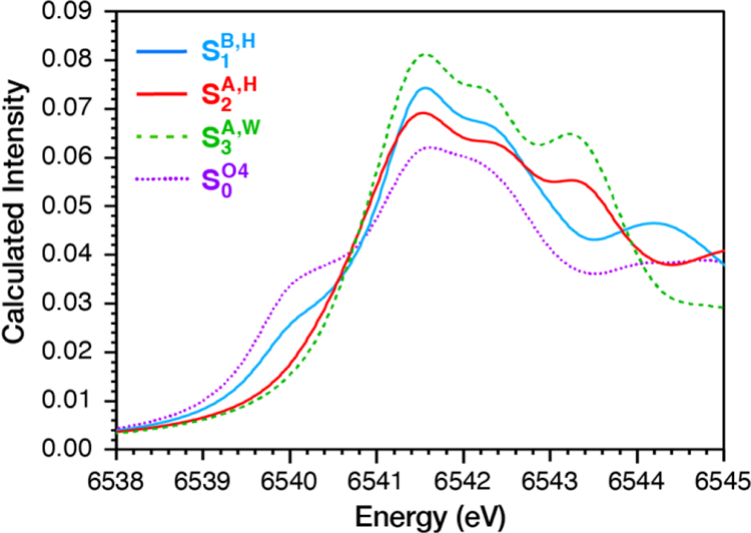
Calculated Mn K-edge XAS spectra for the most experimentally consistent models of states S_0_ to S_3_.

In Figure 8, we show the calculated spectra of the most experimentally consistent models of each state, as identified from the analysis of the difference spectra (structures **S**_**1**_^**B,H**^, **S**_**2**_^**A,H**^, **S**_**3**_^**A,W**^, and **S**_**0**_^**O4**^, as labeled in Figure 5). The most experimentally consistent model of the S_3_ state (**S**_**3**_^**A,W**^) is an all-Mn(IV) model, and hence, the XAS pre-edge region is consistent with Mn-based oxidation in each S-state transition. Importantly, the same model is the one that fully explains observations by electron–electron double resonance (ELDOR) detected nuclear magnetic resonance experiments (EDNMR).^20,21^

The calculated spectra in Figure 5 reproduce all of the major features of the experimental spectra. Specifically, (a) the shoulder at 6539.5 eV is smaller in the S_1_ state than in the S_0_ state and is absent in the S_2_ and S_3_ states, as in experiment; (b) the peak above 6541 eV has the smallest intensity in the S_0_ state and the largest in the S_3_ state; (c) the intensity in the region between 6542 and 6544 eV increases with each S-state transition; and (d) the intersection points of all spectra at 6540.5 and 6544 eV, and of the S_1_ and S_2_ states spectra at 6542.7 eV are also reproduced. Notably, small deviations from experiments in the relative intensities among the S-states are within the accuracy limits of our methodology and can be more easily distinguished in the corresponding difference spectra in Figures 6, 7 and Figure S15.

**Figure 9:**
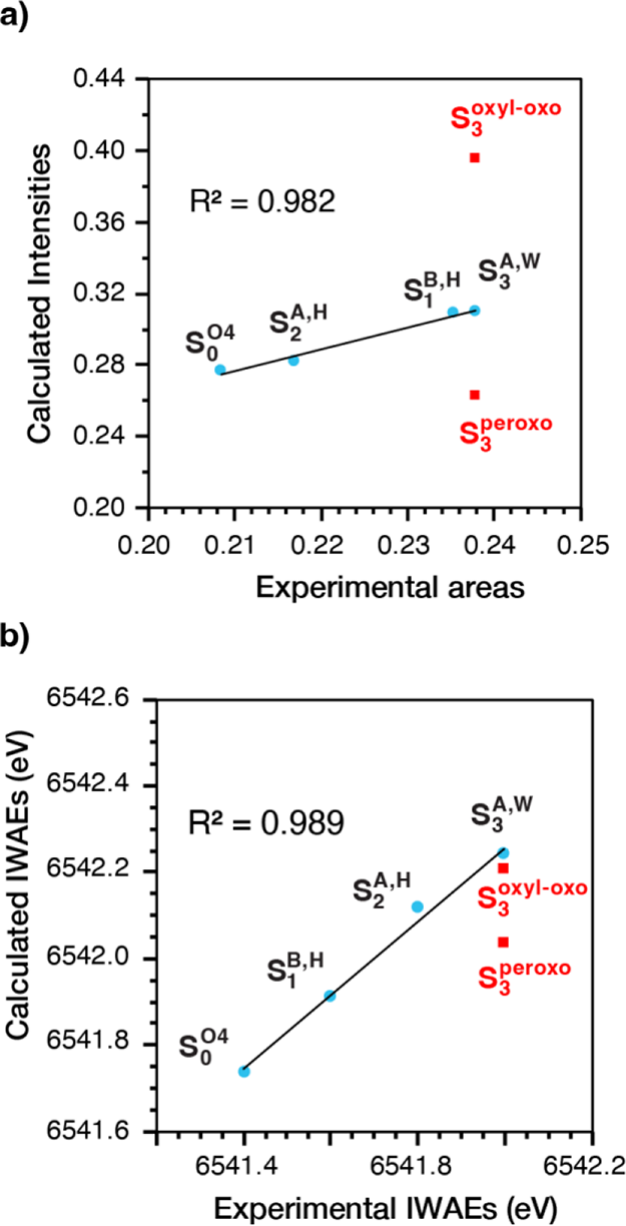
(a) Correlation of summed calculated intensities and experimental pre-edge areas and (b) correlation of calculated and experimental intensity-weighted average energies for different OEC models in different S-states. The correlations show that only Mn-centered oxidation in the S_2_ → S_3_ transition is consistent with the HERFD data.

It is important to note that direct visual comparison of the calculated spectra of a specific state with experimental spectra is not expected to lead to useful conclusions; instead, it is meaningful to compare the total pre-edge area as well as the IWAEs to experiment, as stressed in previous studies.^70,71,96^ Each spectrum results from the distribution of energies and intensities of a vast number of transitions from different Mn ions, and the height and width of each apparent peak of the spectrum are very sensitive to small variations in the relative energies of the transitions it contains. Moreover, it is amply established that errors in the (relative) intensities of the transitions are larger than errors in the energies.^71^ It is precisely for these reasons that the pre-edge area is considered a far better parameter than individual peak heights to assess agreement with experiment.^96^ Indeed, an excellent correlation is observed between summed calculated intensities and experimental pre-edge areas, as presented in Figure 9a. The linear relationship (*R*^2^ = 0.98) shows a strong correspondence between experiment and theory. It also implies that intensity errors are similar among different S-states, which further justifies the use of difference spectra to probe S-state progression. By contrast, the transition intensities of the ligand-based oxidized alternatives for the S_3_ state, **S**_**3**_^**oxyl–oxo**^ and **S**_**3**_^**peroxo**^ (Figure 2), deviate strongly. The computed spectra also reproduce the shift of the IWAEs to higher energy with the S-state progression (Figure 9b).

### Electronic Structure Origin of the Observed Spectral Features

**Figure 10:**
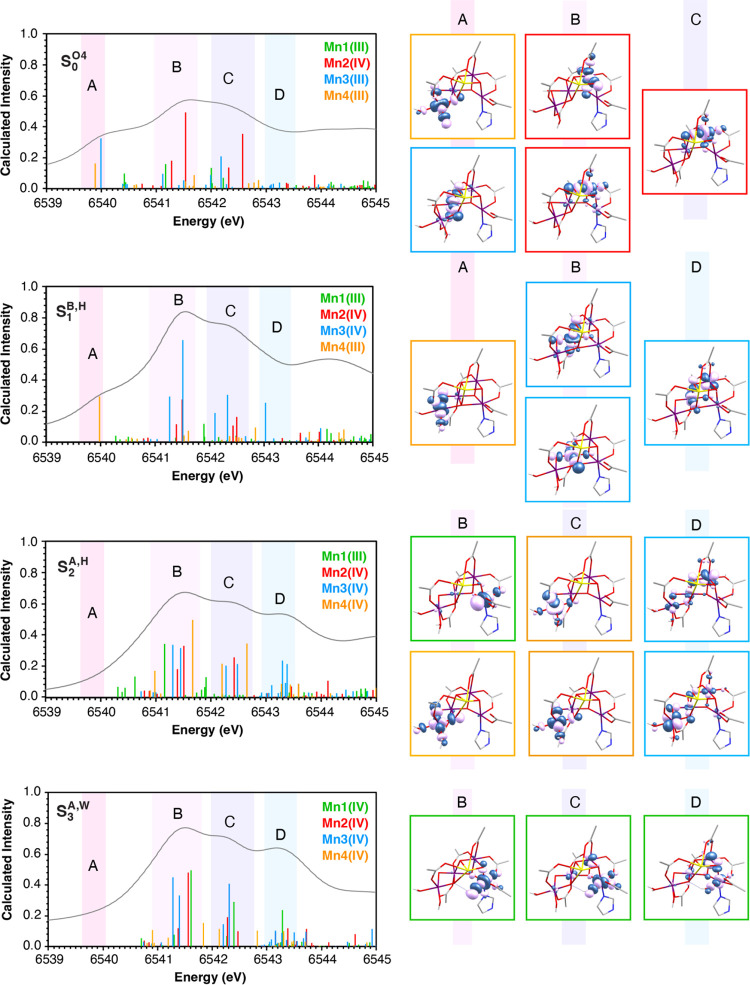
Assignment of the calculated pre-edge XAS spectrum based on the NTOs associated with the transitions for models **S**_**0**_^**O4**^, **S**_**1**_^**B,H**^, **S**_**2**_^**A,H**^, and **S**_**3**_^**A,W**^. In the stick spectra, transitions from 1s core orbitals of Mn1 are shown in green sticks, of Mn2 in red, of Mn3 in blue, and of Mn4 in orange. NTOs for transitions from 1s core orbitals of Mn1 are shown in green boxes, of Mn2 in red, of Mn3 in blue, and of Mn4 in the orange box.

The contributions of each Mn ion to the calculated XAS spectra of the most experimentally consistent models of each S-state are shown in Figure 10, where the stick spectra of each Mn ion are plotted in different colors for each structure. Analysis of the TD-DFT natural transition orbitals (NTOs) reveals the nature of the underlying transitions that form the pre-edge region. The NTOs of **S**_**1**_^**B,H**^, **S**_**2**_^**A,H**^, **S**_**3**_^**A,W**^, and **S**_**0**_^**O4**^ as well as of the redox isomers of the S_3_ state, **S**_**3**_^**A,W**^, **S**_**3**_^**oxyl–oxo**^, and **S**_**3**_^**peroxo**^, are shown in Figures S16–S21. We observe that the nature of the transitions in each energy region remains essentially the same with S-state progression; thus, in Figure 10 we focus on the origins of the differences between successive S-states, and we show the NTOs that contribute the most to those differences.

Specifically, transitions with energy lower than ∼6542 eV are mostly attributed to excitations from the 1s core orbitals to 3d with dominant local Mn character, while transitions with energy higher than 6542 eV also have metal-to-metal charge transfer character. Transitions from the 1s to nonbonding *t*_2*g*_ orbitals have very low intensity due to negligible metal 4p mixing, while transitions to the antibonding d_*z*^2^_ and d_*x*^2^–*y*^2^_ orbitals contribute significantly to the intensity due to increased metal 4p mixing, which is covalently mediated by σ interacting oxygen 2p orbitals. The most intense spectral differences are observed in regions A–D, denoted with pink, purple, and blue hues in the stick spectra plots in Figure 10.

In **S**_**0**_^**O4**^, the peak around 6540 eV (region A) is mostly attributed to local Mn4(III) and Mn3(III) 1s → 3d transitions, whereas in **S**_**1**_^**B,H**^ the latter is blue-shifted due to Mn3(III) oxidation to Mn3(IV), and it appears in region B. In addition, Mn3(IV) transitions in regions B, C, and D gain intensity in **S**_**1**_^**B,H**^, which explains the positive S_1_–S_0_ difference spectra in those regions (Figure S15).

As in the S_0_ → S_1_ transition, Mn4(III) oxidation to Mn4(IV) during the S_1_ → S_2_ transition blue-shifts the local Mn4 1s → 3d transition from region A in **S**_**1**_^**B,H**^ to region B in **S**_**2**_^**A,H**^, and local Mn4(IV) transitions gain intensity in regions B and C. However, the local Mn3(IV) transition at 6541.5 eV has decreased intensity in **S**_**2**_^**A,H**^ relative to **S**_**1**_^**B,H**^, probably due to decreased Mn 3d–4p orbital mixing as a result of coordination geometry changes, leading to negative S_2_–S_1_ difference spectra in region B. Furthermore, a metal-to-metal charge transfer Mn3(IV) 1s → Mn4(IV) 3d transition arises in region D. This qualitative analysis reveals that the effect of metal-based oxidation on the XAS pre-edge region of the OEC is essentially due to increased intensity of transitions that involve the oxidized Mn(IV) ion, consistently with previous reports,^96^ as well as due to coordination geometry changes on the rest of the Mn ions of the cluster.

During the S_2_ → S_3_ transition, Mn1(III) oxidation to Mn1(IV) blue shifts local Mn1 transitions, leading to negative S_3_–S_2_ difference spectra at 6540–6541 eV. Mn1(IV) transitions are more intense in **S**_**3**_^**A,W**^ than **S**_**2**_^**A,H**^ in regions B, C and D, whereas Mn4(IV) transitions are less intense. The effect of those changes is reflected in the calculated S_3_–S_2_ difference spectra, which is in excellent agreement with experiment (Figure 7). Interestingly, the predicted pre-edge regions of the alternative S_3_-state models **S**_**3**_^**oxyl–oxo**^ and **S**_**3**_^**peroxo**^ are strikingly different.

**Figure 11:**
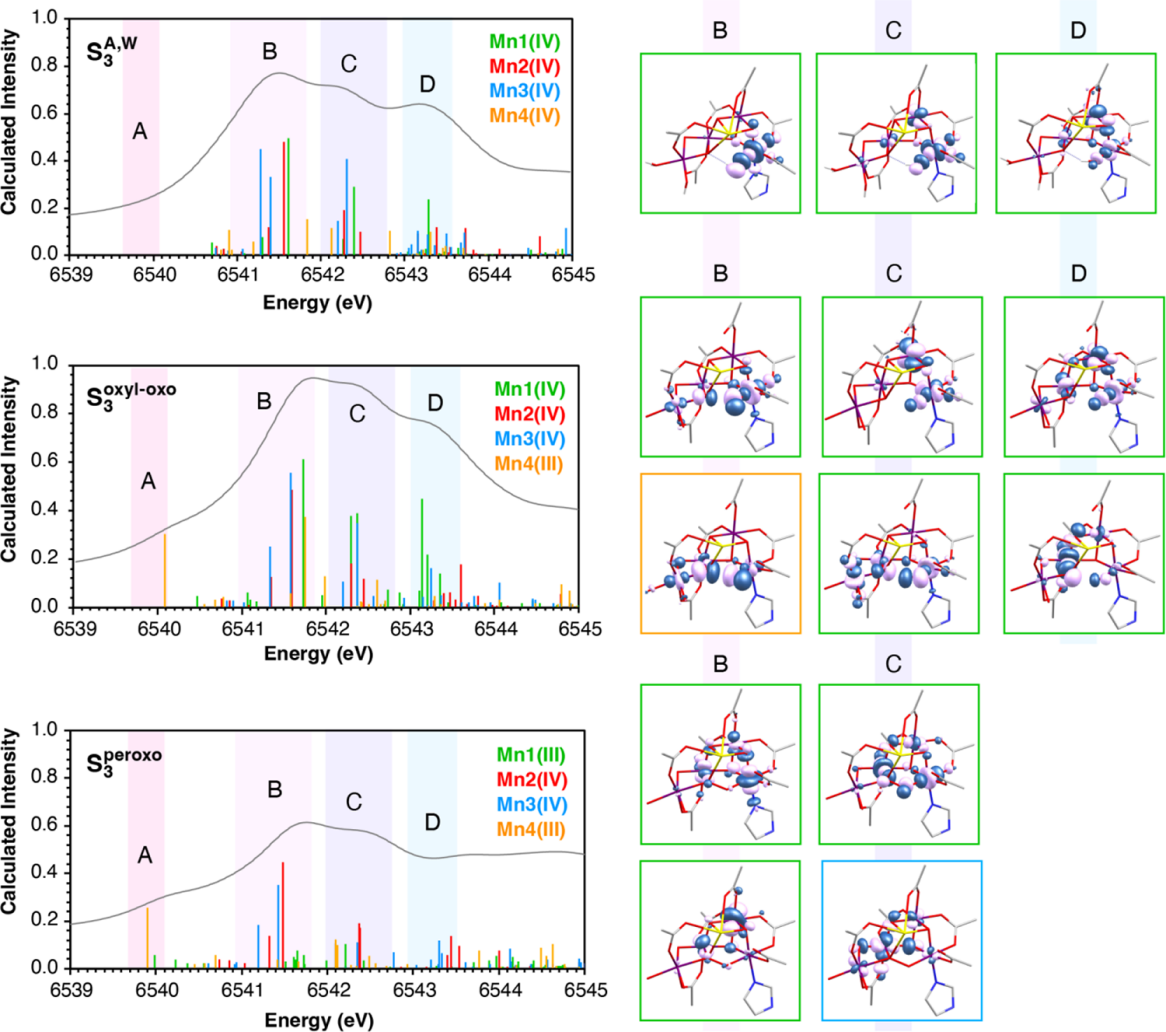
Assignment of the calculated pre-edge XAS spectrum based on the NTOs associated with the transitions for models **S**_**3**_^**A,W**^, **S**_**3**_^**oxyl–oxo**^, and **S**_**3**_^**peroxo**^. In the stick spectra, transitions from 1s core orbitals of Mn1 are shown in green sticks, of Mn2 in red, of Mn3 in blue, and of Mn4 in orange. NTOs for transitions from 1s core orbitals of Mn1 are shown in green boxes, of Mn3 in blue, and of Mn4 in orange box.

The stick spectra of S_3_ state isomers **S**_**3**_^**A,W**^, **S**_**3**_^**oxyl–oxo**^, and **S**_**3**_^**peroxo**^ are compared in Figure 11. Comparison of the stick spectra reveals that the large intensity difference between **S**_**3**_^**oxyl–oxo**^ and **S**_**3**_^**A,W**^ peaks is attributed to regions B, C, and D. Notably, region A has a local Mn4(III) character. The NTOs that correspond to peaks B–D are shown next to each plot in Figure 11. NTOs that correspond to excitations from Mn1 1s core orbitals are shown in green boxes and from Mn4 in the orange box. In addition, the calculated spectra of individual Mn ions are compared in Figure S22. The higher intensity of the transitions of the **S**_**3**_^**oxyl–oxo**^ model compared to the **S**_**3**_^**A,W**^ in the 6541–6544 eV region is attributed mostly to excitations from Mn1 and Mn4 1s core orbitals (Figure S22). Comparison of the NTOs of the B, C and D peaks of **S**_**3**_^**oxyl–oxo**^ and **S**_**3**_^**A,W**^ shows that (a) the local 1s to 3d transitions of Mn1 (first row of **S**_**3**_^**oxyl–oxo**^ NTOs in Figure 11) are more dipole-allowed due to the increased p character of the acceptor orbitals and are thus more intense than the corresponding **S**_**3**_^**A,W**^ peaks, and (b) **S**_**3**_^**oxyl–oxo**^ additionally has three intense charge transfer excitations from Mn1 and Mn4 to O5 and O6 p orbitals (second row of **S**_**3**_^**oxyl–oxo**^ NTOs). Thus, examination of the NTOs of **S**_**3**_^**A,W**^ and **S**_**3**_^**oxyl–oxo**^ shows that the higher calculated intensity for the **S**_**3**_^**oxyl–oxo**^ model is attributed to charge transfer channels enabled by the oxyl-oxo radical.

The lower intensity of the transitions in **S**_**3**_^**peroxo**^ compared to **S**_**3**_^**A,W**^ in the 6541–6544 eV region is traced to the same B, C, and D peaks of **S**_**3**_^**A,W**^ as in the comparison between **S**_**3**_^**A,W**^ and **S**_**3**_^**oxyl–oxo**^. The corresponding peaks in **S**_**3**_^**peroxo**^ have lower intensity, in the case of B and C, or do not exist at all, in the case of D. In the **S**_**3**_^**peroxo**^ acceptor, orbitals of local excitations of Mn1(III) and Mn3(IV) have less p character than the corresponding excitation in **S**_**3**_^**A,W**^, indicating that the peroxo unit hinders charge transfer channels, contrary to the oxyl-oxo and oxo-hydroxo groups.

In summary, a comparison of the experimental and calculated pre-edge XAS spectra supports Mn(III) to Mn(IV) oxidation in each S-state transition. Ligand-based oxidation in the S_2_ → S_3_ transition would result in either significantly more intense (oxyl formation) or significantly less intense (peroxo formation) transitions, therefore this possibility can be safely excluded based on the present HERFD data.

## Conclusions

We have presented HERFD spectra of all S-states of PSII from *T. vestitus*. Spectra were collected as “0, 1, 2, and 3 flashed” samples and deconvoluted to pure S_1_, S_2_, S_3_ and S_0,_ respectively, with the aid of EPR spectroscopy. The energy of the edge shifts to a higher energy during the S_0_ to S_1_ and S_1_ to S_2_ transitions, which clearly reflects Mn-based oxidation. However, the edge shift cannot be used reliably to determine the locus of oxidation during the S_2_ to S_3_ transition, due to inherent complexity of this oxidation reaction as well as due to quantification method limitations. Therefore, we focus on the pre-edge region, leveraging the high-resolution achieved by the HERFD technique and quantum chemical calculations on large DFT-optimized cluster models of the OEC.

The relative intensities of the pre-edge peaks change clearly with the S-state progression. Using quantum mechanical results, we compared different structural variants of each state on the basis of the difference spectra, the sum of intensities, and the IWAEs. Our results demonstrate that this QM-supported analysis of the pre-edge is highly sensitive and can discriminate even between structures with minor differences such as terminal water ligands in different protonation states. The best fitting model for the S_1_ state is one with collinear pseudo-Jahn–Teller axes on Mn1(III) and Mn4(III) ions and with both the W1 and W2 terminal ligands of the Mn4 ion in the aquo form. The S_2_ state is most consistent with an “open-cubane” conformation with an Mn1(III) ion and both Mn4-bound W1 and W2 in the aquo form. In the S_0_ state, protonation of the O4 gives a better fitting with the XAS spectra than protonation of the O5. Crucially, the computed Mn pre-edge spectra are starkly different for distinct variants of the S_3_ state and support exclusively the Mn(IV)_4_ oxo-hydroxo formulation as the species observed experimentally, excluding the Mn(IV)_3_Mn(III) oxyl-oxo and Mn(IV)_2_Mn(III)_2_ peroxo models. Electronic structure analysis shows that if an oxyl-oxo group were present, the pre-edge intensity would be significantly higher because it would enable charge-transfer channels from the 1s core orbitals of Mn1 and Mn4 to the corresponding valence 3d orbitals, which would have higher p mixing. By contrast, the presence of a peroxo group in the S_3_ state would have exactly the opposite effect on the transition intensities, due to blocking of Mn1 and Mn3 charge transfer transitions attributed to lower p mixing. Importantly, this is fully consistent with magnetic resonance data, where the manganese hyperfine tensors are all nearly isotropic, which is only possible if they are all Mn(IV).^20^

By combining high-resolution XAS data with QM calculations, we have clearly shown that recent controversies regarding the nature of the S_3_ state can be rigorously resolved and that presence of either peroxo or oxo/oxyl level intermediate can be ruled out. An all-Mn(IV) S_3_ state should be considered as the starting intermediate in the interpretation of experimental data on the S_3_ to S_0_ transition.^97,98^ Overall, the results of this work clearly show Mn-based oxidation during the S_2_ to S_3_ transition and constrain the possible mechanisms of the formation of the O–O bond to those that initiate after the final light-driven oxidation. Beyond the importance for biological water oxidation, the cumulative metal-centered storage of oxidizing equivalents supported by the present study directs our attention to synthetic water oxidation catalysts that leverage multimetallic cooperativity.

## Supporting Information Available

Additional experimental details; representative EPR spectra and experimental multiline signal intensity after 1–5 flashes (Figure S1); calculated multiline intensity in comparison with the experimental using different combinations of fitting parameters (Table S1); calculated S state content after a given number of flashes (Table S2); inhomogeneity of the S_2_ and S_3_ states as indicated by X-band and W-band EPR spectroscopy (Figure S2); damage test in 0, 1, 2, 3 flashed samples (Figure S3); standard error of the 0, 1, 2, 3 flashed spectra and of the pure S_1_, S_2_, S_3_, S_0_ spectra (Figure S4); QM model of the S_1_ state (Figure S5); first derivative of the XANES spectra (Figure S6); second derivative of the XANES spectra (Figure S7); HERFD spectra of this work in comparison with literature data (Figure S8 and S9); fitting of the pre-edge experimental peaks with Voigt curves (Figure S10); parameters of the fitting of the pre-edge region (Table S3); standard error of the S_*i*_–S_*i*-1_ differences (Figure S11); schematic depiction of the inorganic cores of all optimized S_0_–S_3_ models (Figure S12); calculated XAS spectra of all models (Figure S13); comparison of calculated S_2_–S_1_ difference spectra for models **S**_**1**_^**B,H**^ and **S**_**2**_^**A,H**^ to those for models **S**_**1**_^**B,H**^ and a mixture of 80% **S**_**2**_^**A,H**^ and 20% **S**_**2**_^**B,H**^ (Figure S14); experimental and calculated S_1_–S_0_ spectral differences for different of S_0_ and S_1_ isomers (Figure S15); assignment of the calculated pre-edge XAS spectra based on NTOs for models **S**_**1**_^**B**^ and **S**_**1**_^**B,H**^ (Figure S16), model **S**_**2**_^**A**^ (Figure S17), model **S**_**3**_^**A,W**^ (Figure S18), model **S**_**3**_^**oxyl–oxo**^ (Figure S19), model **S**_**3**_^**peroxo**^ (Figure S20), and model **S**_**0**_^**O4**^ (Figure S21); calculated XAS spectra for individual Mn ions for **S**_**3**_^**A,W**^, **S**_**3**_^**B,W**^, **S**_**3**_^**oxyl–oxo**^, and **S**_**3**_^**peroxo**^ (Figure S22); example of ORCA input file (PDF)
Cartesian coordinates of all structural models discussed in this work (TXT)

## References

[ref1] Blankenship R. E. Molecular mechanisms of photosynthesis; 3rd ed.; John Wiley & Sons: Chichester, 2021; p 352.

[ref2] Shevela D.; Björn L. O. Photosynthesis: solar energy for life; World Scientific Publishing: Singapore, 2018; p 204 10.1142/10522.

[ref3] Yano J.; Yachandra V. Mn_4_Ca Cluster in Photosynthesis: Where and How Water is Oxidized to Dioxygen. Chem. Rev. 2014, 114, 4175–4205. 10.1021/cr4004874. 24684576 PMC4002066

[ref4] Shen J.-R. The Structure of Photosystem II and the Mechanism of Water Oxidation in Photosynthesis. Annu. Rev. Plant Biol. 2015, 66, 23–48. 10.1146/annurev-arplant-050312-120129. 25746448

[ref5] Shevela D.; Kern J. F.; Govindjee G.; Messinger J. Solar energy conversion by photosystem II: principles and structures. Photosynth. Res. 2023, 156, 279–307. 10.1007/s11120-022-00991-y. 36826741 PMC10203033

[ref6] Kulik L. V.; Epel B.; Lubitz W.; Messinger J. ^55^Mn Pulse ENDOR at 34 GHz of the S_0_ and S_2_ States of the Oxygen-Evolving Complex in Photosystem II. J. Am. Chem. Soc. 2005, 127, 2392–2393. 10.1021/ja043012j. 15724984

[ref7] Kulik L. V.; Epel B.; Lubitz W.; Messinger J. Electronic Structure of the Mn_4_O_x_Ca Cluster in the S_0_ and S_2_ States of the Oxygen-Evolving Complex of Photosystem II Based on Pulse ^55^Mn-ENDOR and EPR Spectroscopy. J. Am. Chem. Soc. 2007, 129, 13421–13435. 10.1021/ja071487f. 17927172

[ref8] Krewald V.; Retegan M.; Cox N.; Messinger J.; Lubitz W.; DeBeer S.; Neese F.; Pantazis D. A. Metal oxidation states in biological water splitting. Chem. Sci. 2015, 6, 1676–1695. 10.1039/C4SC03720K. 29308133 PMC5639794

[ref9] Zaharieva I.; Chernev P.; Berggren G.; Anderlund M.; Styring S.; Dau H.; Haumann M. Room-Temperature Energy-Sampling Kβ X-ray Emission Spectroscopy of the Mn_4_Ca Complex of Photosynthesis Reveals Three Manganese-Centered Oxidation Steps and Suggests a Coordination Change Prior to O_2_ Formation. Biochemistry 2016, 55, 4197–4211. 10.1021/acs.biochem.6b00491. 27377097

[ref10] Schuth N.; Zaharieva I.; Chernev P.; Berggren G.; Anderlund M.; Styring S.; Dau H.; Haumann M. K_α_ X-ray Emission Spectroscopy on the Photosynthetic Oxygen-Evolving Complex Supports Manganese Oxidation and Water Binding in the S_3_ State. Inorg. Chem. 2018, 57, 10424–10430. 10.1021/acs.inorgchem.8b01674. 30067343

[ref11] Cheah M. H.; Zhang M.; Shevela D.; Mamedov F.; Zouni A.; Messinger J. Assessment of the manganese cluster’s oxidation state via photoactivation of photosystem II microcrystals. Proc. Natl. Acad. Sci. U. S. A. 2020, 117, 141–145. 10.1073/pnas.1915879117. 31848244 PMC6955365

[ref12] Pantazis D. A. The S_3_ State of the Oxygen-Evolving Complex: Overview of Spectroscopy and XFEL Crystallography with a Critical Evaluation of Early-Onset Models for O–O Bond Formation. Inorganics 2019, 7, 55 10.3390/inorganics7040055.

[ref13] Pantazis D. A. Missing Pieces in the Puzzle of Biological Water Oxidation. ACS Catal. 2018, 8, 9477–9507. 10.1021/acscatal.8b01928.

[ref14] Renger G. Mechanistic and structural aspects of photosynthetic water oxidation. Physiol. Plant. 1997, 100, 828–841. 10.1111/j.1399-3054.1997.tb00009.x.

[ref15] Messinger J.; Robblee J. H.; Bergmann U.; Fernandez C.; Glatzel P.; Visser H.; Cinco R. M.; McFarlane K. L.; Bellacchio E.; Pizarro S. A.; Cramer S. P.; Sauer K.; Klein M. P.; Yachandra V. K. Absence of Mn-centered oxidation in the S_2_ → S_3_ transition: implications for the mechanism of photosynthetic water oxidation. J. Am. Chem. Soc. 2001, 123, 7804–7820. 10.1021/ja004307+. 11493054 PMC3965774

[ref16] Renger G. Coupling of electron and proton transfer in oxidative water cleavage in photosynthesis. Biochim. Biophys. Acta Bioenerg. 2004, 1655, 195–204. 10.1016/j.bbabio.2003.07.007. 15100032

[ref17] Haumann M.; Muller C.; Liebisch P.; Iuzzolino L.; Dittmer J.; Grabolle M.; Neisius T.; Meyer-Klaucke W.; Dau H. Structural and oxidation state changes of the photosystem II manganese complex in four transitions of the water oxidation cycle (S_0_ → S_1_, S_1_ → S_2_, S_2_ → S_3_, and S_3,4_ → S_0_) characterized by X-ray absorption spectroscopy at 20 K and room temperature. Biochemistry 2005, 44, 1894–1908. 10.1021/bi048697e. 15697215

[ref18] Renger G. Oxidative photosynthetic water splitting: energetics, kinetics and mechanism. Photosynth. Res. 2007, 92, 407–425. 10.1007/s11120-007-9185-x. 17647091

[ref19] Boussac A.; Sugiura M.; Rutherford A. W.; Dorlet P. Complete EPR Spectrum of the S_3_-State of the Oxygen-Evolving Photosystem II. J. Am. Chem. Soc. 2009, 131, 5050–5051. 10.1021/ja900680t. 19320479

[ref20] Cox N.; Retegan M.; Neese F.; Pantazis D. A.; Boussac A.; Lubitz W. Electronic structure of the oxygen-evolving complex in photosystem II prior to O–O bond formation. Science 2014, 345, 804–808. 10.1126/science.1254910. 25124437

[ref21] Marchiori D. A.; Debus R. J.; Britt R. D. Pulse EPR Spectroscopic Characterization of the S_3_ State of the Oxygen-Evolving Complex of Photosystem II Isolated from *Synechocystis*. Biochemistry 2020, 59, 4864–4872. 10.1021/acs.biochem.0c00880. 33319991

[ref22] Chrysina M.; Heyno E.; Kutin Y.; Reus M.; Nilsson H.; Nowaczyk M. M.; DeBeer S.; Neese F.; Messinger J.; Lubitz W.; Cox N. Five-coordinate Mn(IV) intermediate in the activation of nature’s water splitting cofactor. Proc. Natl. Acad. Sci. U. S. A. 2019, 116, 16841–16846. 10.1073/pnas.1817526116. 31391299 PMC6708314

[ref23] Zahariou G.; Ioannidis N.; Sanakis Y.; Pantazis D. A. Arrested Substrate Binding Resolves Catalytic Intermediates in Higher-Plant Water Oxidation. Angew. Chem., Int. Ed. 2021, 60, 3156–3162. 10.1002/anie.202012304. PMC7898718 33030775

[ref24] Sanakis Y.; Sarrou J.; Zahariou G.; Petrouleas V. In Photosynthesis. Energy from the Sun; Allen J. F.; Gantt E.; Golbeck J. H.; Osmond B., Eds.; Springer Netherlands: Dordrecht, 2008; p 479–482.

[ref25] Retegan M.; Krewald V.; Mamedov F.; Neese F.; Lubitz W.; Cox N.; Pantazis D. A. A five-coordinate Mn(IV) intermediate in biological water oxidation: spectroscopic signature and a pivot mechanism for water binding. Chem. Sci. 2016, 7, 72–84. 10.1039/C5SC03124A. 29861966 PMC5950799

[ref26] Davis K. M.; Sullivan B. T.; Palenik M. C.; Yan L.; Purohit V.; Robison G.; Kosheleva I.; Henning R. W.; Seidler G. T.; Pushkar Y. Rapid Evolution of the Photosystem II Electronic Structure during Water Splitting. Phys. Rev. X 2018, 8, 041014 10.1103/PhysRevX.8.041014. 31231592 PMC6588194

[ref27] Suga M.; Akita F.; Sugahara M.; Kubo M.; Nakajima Y.; Nakane T.; Yamashita K.; Umena Y.; Nakabayashi M.; Yamane T.; Nakano T.; Suzuki M.; Masuda T.; Inoue S.; Kimura T.; Nomura T.; Yonekura S.; Yu L. J.; Sakamoto T.; Motomura T.; Chen J. H.; Kato Y.; Noguchi T.; Tono K.; Joti Y.; Kameshima T.; Hatsui T.; Nango E.; Tanaka R.; Naitow H.; Matsuura Y.; Yamashita A.; Yamamoto M.; Nureki O.; Yabashi M.; Ishikawa T.; Iwata S.; Shen J. R. Light-induced structural changes and the site of O = O bond formation in PSII caught by XFEL. Nature 2017, 543, 131–135. 10.1038/nature21400. 28219079

[ref28] Suga M.; Akita F.; Yamashita K.; Nakajima Y.; Ueno G.; Li H.; Yamane T.; Hirata K.; Umena Y.; Yonekura S.; Yu L.-J.; Murakami H.; Nomura T.; Kimura T.; Kubo M.; Baba S.; Kumasaka T.; Tono K.; Yabashi M.; Isobe H.; Yamaguchi K.; Yamamoto M.; Ago H.; Shen J.-R. An oxyl/oxo mechanism for oxygen-oxygen coupling in PSII revealed by an X-ray free-electron laser. Science 2019, 366, 334–338. 10.1126/science.aax6998. 31624207

[ref29] Kern J.; Chatterjee R.; Young I. D.; Fuller F. D.; Lassalle L.; Ibrahim M.; Gul S.; Fransson T.; Brewster A. S.; Alonso-Mori R.; Hussein R.; Zhang M.; Douthit L.; de Lichtenberg C.; Cheah M. H.; Shevela D.; Wersig J.; Seuffert I.; Sokaras D.; Pastor E.; Weninger C.; Kroll T.; Sierra R. G.; Aller P.; Butryn A.; Orville A. M.; Liang M.; Batyuk A.; Koglin J. E.; Carbajo S.; Boutet S.; Moriarty N. W.; Holton J. M.; Dobbek H.; Adams P. D.; Bergmann U.; Sauter N. K.; Zouni A.; Messinger J.; Yano J.; Yachandra V. K. Structures of the intermediates of Kok’s photosynthetic water oxidation clock. Nature 2018, 563, 421–425. 10.1038/s41586-018-0681-2. 30405241 PMC6485242

[ref30] Ibrahim M.; Fransson T.; Chatterjee R.; Cheah M. H.; Hussein R.; Lassalle L.; Sutherlin K. D.; Young I. D.; Fuller F. D.; Gul S.; Kim I.-S.; Simon P. S.; de Lichtenberg C.; Chernev P.; Bogacz I.; Pham C. C.; Orville A. M.; Saichek N.; Northen T.; Batyuk A.; Carbajo S.; Alonso-Mori R.; Tono K.; Owada S.; Bhowmick A.; Bolotovsky R.; Mendez D.; Moriarty N. W.; Holton J. M.; Dobbek H.; Brewster A. S.; Adams P. D.; Sauter N. K.; Bergmann U.; Zouni A.; Messinger J.; Kern J.; Yachandra V. K.; Yano J. Untangling the sequence of events during the S_2_ → S_3_ transition in photosystem II and implications for the water oxidation mechanism. Proc. Natl. Acad. Sci. U. S. A. 2020, 117, 12624–12635. 10.1073/pnas.2000529117. 32434915 PMC7293653

[ref31] Simon P. S.; Makita H.; Bogacz I.; Fuller F.; Bhowmick A.; Hussein R.; Ibrahim M.; Zhang M.; Chatterjee R.; Cheah M. H.; Chernev P.; Doyle M. D.; Brewster A. S.; Alonso-Mori R.; Sauter N. K.; Bergmann U.; Dobbek H.; Zouni A.; Messinger J.; Kern J.; Yachandra V. K.; Yano J. Capturing the sequence of events during the water oxidation reaction in photosynthesis using XFELs. FEBS Lett. 2023, 597, 30–37. 10.1002/1873-3468.14527. 36310373 PMC9839502

[ref32] Corry T. A.; O’Malley P. J. Evidence of O–O Bond Formation in the Final Metastable S_3_ State of Nature’s Water Oxidizing Complex Implying a Novel Mechanism of Water Oxidation. J. Phys. Chem. Lett. 2018, 9, 6269–6274. 10.1021/acs.jpclett.8b02793. 30336040

[ref33] Yamaguchi K.; Miyagawa K.; Shoji M.; Isobe H.; Kawakami T. Elucidation of a multiple S_3_ intermediates model for water oxidation in the oxygen evolving complex of photosystem II. Calcium-assisted concerted O-O bond formation. Chem. Phys. Lett. 2022, 806, 140042 10.1016/j.cplett.2022.140042.

[ref34] Drosou M.; Comas-Vila G.; Neese F.; Salvador P.; Pantazis D. A. Does Serial Femtosecond Crystallography Depict State-Specific Catalytic Intermediates of the Oxygen-Evolving Complex?. J. Am. Chem. Soc. 2023, 145, 10604–10621. 10.1021/jacs.3c00489. 37137865 PMC10197136

[ref35] Tanaka A.; Fukushima Y.; Kamiya N. Two Different Structures of the Oxygen-Evolving Complex in the Same Polypeptide Frameworks of Photosystem II. J. Am. Chem. Soc. 2017, 139, 1718–1721. 10.1021/jacs.6b09666. 28102667

[ref36] Renger G. Mechanism of light induced water splitting in Photosystem II of oxygen evolving photosynthetic organisms. Biochim. Biophys. Acta Bioenerg. 2012, 1817, 1164–1176. 10.1016/j.bbabio.2012.02.005. 22353626

[ref37] Isobe H.; Shoji M.; Shen J.-R.; Yamaguchi K. Chemical Equilibrium Models for the S_3_ State of the Oxygen-Evolving Complex of Photosystem II. Inorg. Chem. 2016, 55, 502–511. 10.1021/acs.inorgchem.5b02471. 26717045

[ref38] Pushkar Y.; Davis K. M.; Palenik M. C. Model of the Oxygen Evolving Complex Which Is Highly Predisposed to O–O Bond Formation. J. Phys. Chem. Lett. 2018, 9, 3525–3531. 10.1021/acs.jpclett.8b00800. 29863871

[ref39] Isobe H.; Shoji M.; Suzuki T.; Shen J.-R.; Yamaguchi K. Spin, Valence, and Structural Isomerism in the S _3_ State of the Oxygen-Evolving Complex of Photosystem II as a Manifestation of Multimetallic Cooperativity. J. Chem. Theory Comput. 2019, 15, 2375–2391. 10.1021/acs.jctc.8b01055. 30855953

[ref40] Corry T. A.; O’Malley P. J. Electronic–Level View of O–O Bond Formation in Nature’s Water Oxidizing Complex. J. Phys. Chem. Lett. 2020, 11, 4221–4225. 10.1021/acs.jpclett.0c00794. 32374174

[ref41] Drosou M.; Pantazis D. A. Redox Isomerism in the S_3_ State of the Oxygen-Evolving Complex Resolved by Coupled Cluster Theory. Chem.—Eur. J. 2021, 27, 12815–12825. 10.1002/chem.202101567. 34288176 PMC8518824

[ref99] Rummel F.; O’Malley P. J. How Nature Makes O2: an Electronic Level Mechanism for Water Oxidation in Photosynthesis. J. Phys. Chem. B 2022, 126, 8214–8221. 10.1021/acs.jpcb.2c06374. 36206029 PMC9589598

[ref42] Krewald V.; Neese F.; Pantazis D. A. Implications of structural heterogeneity for the electronic structure of the final oxygen-evolving intermediate in photosystem II. J. Inorg. Biochem. 2019, 199, 110797 10.1016/j.jinorgbio.2019.110797. 31404888

[ref43] Glöckner C.; Kern J.; Broser M.; Zouni A.; Yachandra V.; Yano J. Structural Changes of the Oxygen-evolving Complex in Photosystem II during the Catalytic Cycle. J. Biol. Chem. 2013, 288, 22607–22620. 10.1074/jbc.M113.476622. 23766513 PMC3829347

[ref44] Chrysina M.; Heyno E.; Kutin Y.; Reus M.; Nilsson H.; Nowaczyk M. M.; DeBeer S.; Neese F.; Messinger J.; Lubitz W.; Cox N. Five-coordinate MnIV intermediate in the activation of nature’s water splitting cofactor. Proc. Natl. Acad. Sci. U. S. A. 2019, 116, 16841–16846. 10.1073/pnas.1817526116. 31391299 PMC6708314

[ref45] Chatterjee R.; Han G.; Kern J.; Gul S.; Fuller F. D.; Garachtchenko A.; Young I. D.; Weng T.-C.; Nordlund D.; Alonso-Mori R.; Bergmann U.; Sokaras D.; Hatakeyama M.; Yachandra V. K.; Yano J. Structural changes correlated with magnetic spin state isomorphism in the S _2_ state of the Mn _4_ CaO _5_ cluster in the oxygen-evolving complex of photosystem II. Chem. Sci. 2016, 7, 5236–5248. 10.1039/C6SC00512H. 28044099 PMC5201215

[ref46] Kuhl H.; Kruip J.; Seidler A.; Krieger-Liszkay A.; Bunker M.; Bald D.; Scheidig A. J.; Rogner M. Towards structural determination of the water-splitting enzyme. Purification, crystallization, and preliminary crystallographic studies of photosystem II from a thermophilic cyanobacterium. J. Biol. Chem. 2000, 275, 20652–20659. 10.1074/jbc.M001321200. 10748017

[ref47] Shinkarev V. P.; Wraight C. A. Oxygen evolution in photosynthesis: from unicycle to bicycle. Proc. Natl. Acad. Sci. U. S. A. 1993, 90, 1834–1838. 10.1073/pnas.90.5.1834. 11607372 PMC45974

[ref48] de Wijn R.; van Gorkom H. J. S-state dependence of the miss probability in Photosystem II. Photosynth. Res. 2002, 72, 217–222. 10.1023/A:1016128632704. 16228520

[ref49] Hillier W.; Messinger J. In Photosystem II: The Light-Driven Water:Plastoquinone Oxidoreductase; Wydrzynski T. J.; Satoh K.; Freeman J. A., Eds.; Springer Netherlands: Dordrecht, 2005; p 567–608 10.1007/1-4020-4254-X.

[ref50] Messinger J.; Renger G. In Primary Processes of Photosynthesis, Part 2: Principles and Apparatus; The Royal Society of Chemistry: Cambridge, 2008; Vol. 9, p 291–349.

[ref51] Han G.; Mamedov F.; Styring S. Misses during Water Oxidation in Photosystem II Are S State-dependent. J. Biol. Chem. 2012, 287, 13422–13429. 10.1074/jbc.M112.342543. 22374999 PMC3339926

[ref52] Suzuki H.; Sugiura M.; Noguchi T. Determination of the Miss Probabilities of Individual S-State Transitions during Photosynthetic Water Oxidation by Monitoring Electron Flow in Photosystem II Using FTIR Spectroscopy. Biochemistry 2012, 51, 6776–6785. 10.1021/bi300708a. 22880689

[ref53] Pham L. V.; Messinger J. Probing S-state advancements and recombination pathways in photosystem II with a global fit program for flash-induced oxygen evolution pattern. Biochim. Biophys. Acta Bioenerg. 2016, 1857, 848–859. 10.1016/j.bbabio.2016.03.013. 27033305

[ref54] Pham L. V.; Janna Olmos J. D.; Chernev P.; Kargul J.; Messinger J. Unequal misses during the flash-induced advancement of photosystem II: effects of the S state and acceptor side cycles. Photosynth. Res. 2019, 139, 93–106. 10.1007/s11120-018-0574-0. 30191436 PMC6373315

[ref55] Han G.; Chernev P.; Styring S.; Messinger J.; Mamedov F. Molecular basis for turnover inefficiencies (misses) during water oxidation in photosystem II. Chem. Sci. 2022, 13, 8667–8678. 10.1039/D2SC00854H. 35974765 PMC9337725

[ref56] Sokaras D.; Weng T. C.; Nordlund D.; Alonso-Mori R.; Velikov P.; Wenger D.; Garachtchenko A.; George M.; Borzenets V.; Johnson B.; Rabedeau T.; Bergmann U. A seven-crystal Johann-type hard x-ray spectrometer at the Stanford Synchrotron Radiation Lightsource. Rev. Sci. Instrum. 2013, 84, 053102 10.1063/1.4803669. 23742527 PMC4108715

[ref57] Neese F.; Wennmohs F.; Becker U.; Riplinger C. The ORCA quantum chemistry program package. J. Chem. Phys. 2020, 152, 224108 10.1063/5.0004608. 32534543

[ref58] Becke A. D. Density-functional thermochemistry. III. The role of exact exchange. J. Chem. Phys. 1993, 98, 5648–5652. 10.1063/1.464913.

[ref59] Lee C.; Yang W.; Parr R. G. Development of the Colle-Salvetti correlation-energy formula into a functional of the electron density. Phys. Rev. B 1988, 37, 785–789. 10.1103/PhysRevB.37.785. 9944570

[ref60] Caldeweyher E.; Ehlert S.; Hansen A.; Neugebauer H.; Spicher S.; Bannwarth C.; Grimme S. A generally applicable atomic-charge dependent London dispersion correction. J. Chem. Phys. 2019, 150, 154122 10.1063/1.5090222. 31005066

[ref61] Lenthe E. v.; Baerends E. J.; Snijders J. G. Relativistic regular two-component Hamiltonians. J. Chem. Phys. 1993, 99, 4597–4610. 10.1063/1.466059.

[ref62] van Lenthe E.; Baerends E. J.; Snijders J. G. Relativistic total energy using regular approximations. J. Chem. Phys. 1994, 101, 9783–9792. 10.1063/1.467943.

[ref63] van Wüllen C. Molecular density functional calculations in the regular relativistic approximation: Method, application to coinage metal diatomics, hydrides, fluorides and chlorides, and comparison with first-order relativistic calculations. J. Chem. Phys. 1998, 109, 392–399. 10.1063/1.476576.

[ref64] Pantazis D. A.; Chen X.-Y.; Landis C. R.; Neese F. All-Electron Scalar Relativistic Basis Sets for Third-Row Transition Metal Atoms. J. Chem. Theory Comput. 2008, 4, 908–919. 10.1021/ct800047t. 26621232

[ref65] Weigend F.; Ahlrichs R. Balanced basis sets of split valence, triple zeta valence and quadruple zeta valence quality for H to Rn: Design and assessment of accuracy. Phys. Chem. Chem. Phys. 2005, 7, 3297–3305. 10.1039/b508541a. 16240044

[ref66] Barone V.; Cossi M. Quantum Calculation of Molecular Energies and Energy Gradients in Solution by a Conductor Solvent Model. J. Phys. Chem. A 1998, 102, 1995–2001. 10.1021/jp9716997.

[ref67] Hirata S.; Head-Gordon M. Time-dependent density functional theory within the Tamm–Dancoff approximation. Chem. Phys. Lett. 1999, 314, 291–299. 10.1016/S0009-2614(99)01149-5.

[ref68] Neese F.; Olbrich G. Efficient use of the resolution of the identity approximation in time-dependent density functional calculations with hybrid density functionals. Chem. Phys. Lett. 2002, 362, 170–178. 10.1016/S0009-2614(02)01053-9.

[ref69] Staroverov V. N.; Scuseria G. E.; Tao J.; Perdew J. P. Comparative assessment of a new nonempirical density functional: Molecules and hydrogen-bonded complexes. J. Chem. Phys. 2003, 119, 12129–12137. 10.1063/1.1626543.

[ref70] Krewald V.; Lassalle-Kaiser B.; Boron T. T. III; Pollock C. J.; Kern J.; Beckwith M. A.; Yachandra V. K.; Pecoraro V. L.; Yano J.; Neese F.; DeBeer S. The Protonation States of Oxo-Bridged MnIV Dimers Resolved by Experimental and Computational Mn K Pre-Edge X-ray Absorption Spectroscopy. Inorg. Chem. 2013, 52, 12904–12914. 10.1021/ic4008203. 24161030 PMC3911776

[ref71] Roemelt M.; Beckwith M. A.; Duboc C.; Collomb M.-N.; Neese F.; DeBeer S. Manganese K-Edge X-Ray Absorption Spectroscopy as a Probe of the Metal–Ligand Interactions in Coordination Compounds. Inorg. Chem. 2012, 51, 680–687. 10.1021/ic202229b. 22145735

[ref72] Neese F.; Wennmohs F.; Hansen A. Efficient and accurate local approximations to coupled-electron pair approaches: An attempt to revive the pair natural orbital method. J. Chem. Phys. 2009, 130, 114108 10.1063/1.3086717. 19317532

[ref73] Dau H.; Liebisch P.; Haumann M. The manganese complex of oxygenic photosynthesis: conversion of five-coordinated Mn(III) to six-coordinated Mn(IV) in the S_2_-S_3_ transition is implied by XANES simulations. Phys. Scr. 2005, 2005, 844 10.1238/Physica.Topical.115a00844.

[ref74] Iuzzolino L.; Dittmer J.; Dörner W.; Meyer-Klaucke W.; Dau H. X-ray Absorption Spectroscopy on Layered Photosystem II Membrane Particles Suggests Manganese-Centered Oxidation of the Oxygen-Evolving Complex for the S_0_-S_1_, S_1_-S_2_, and S_2_-S_3_ Transitions of the Water Oxidation Cycle. Biochemistry 1998, 37, 17112–17119. 10.1021/bi9817360. 9860823

[ref75] Dittmer J.; Iuzzolino L.; Dörner W.; Nolting H. F.; Meyer-Klaucke W.; Dau H. Photosynthesis: Mechanisms and Effects: Vol. I–V: Proceedings of the XIth International Congress on Photosynthesis, Budapest, Hungary, August 17–22, 1998; Garab G., Ed.; Springer Netherlands: Dordrecht, 1998; p 1339–1342 10.1007/978-94-011-3953-3.

[ref76] Haumann M.; Liebisch P.; Muller C.; Barra M.; Grabolle M.; Dau H. Photosynthetic O2 formation tracked by time-resolved x-ray experiments. Science 2005, 310, 1019–1021. 10.1126/science.1117551. 16284178

[ref77] Roelofs T. A.; Liang W.; Latimer M. J.; Cinco R. M.; Rompel A.; Andrews J. C.; Sauer K.; Yachandra V. K.; Klein M. P. Oxidation states of the manganese cluster during the flash-induced S-state cycle of the photosynthetic oxygen-evolving complex. Proc. Natl. Acad. Sci. U. S. A. 1996, 93, 3335–3340. 10.1073/pnas.93.8.3335. 11607649 PMC39608

[ref78] Isobe H.; Shoji M.; Suzuki T.; Shen J.-R.; Yamaguchi K. Exploring reaction pathways for the structural rearrangements of the Mn cluster induced by water binding in the S_3_ state of the oxygen evolving complex of photosystem II. J. Photochem. Photobiol. A: Chem. 2021, 405, 112905 10.1016/j.jphotochem.2020.112905.

[ref79] Isobe H.; Shoji M.; Suzuki T.; Shen J.-R.; Yamaguchi K. Roles of the Flexible Primary Coordination Sphere of the Mn_4_CaO_x_ Cluster: What Are the Immediate Decay Products of the S_3_ State?. J. Phys. Chem. B 2022, 126, 7212–7228. 10.1021/acs.jpcb.2c02596. 36107406

[ref80] Miyagawa K.; Shoji M.; Isobe H.; Kawakami T.; Nakajima T.; Yamaguchi K. Relative energies among S_3_ intermediates in the photosystem II revealed by DLPNO coupled cluster and hybrid DFT calculations. Possible pathways of water insertion in the S_2_ to S_3_ transition. Chem. Phys. Lett. 2022, 793, 139439 10.1016/j.cplett.2022.139439.

[ref81] Yamaguchi K.; Miyagawa K.; Shoji M.; Isobe H.; Kawakami T. Elucidation of a multiple S3 intermediates model for water oxidation in the oxygen evolving complex of photosystem II. Calcium-assisted concerted OO bond formation. Chem. Phys. Lett. 2022, 806, 140042 10.1016/j.cplett.2022.140042.

[ref82] Glatzel P.; Schroeder H.; Pushkar Y.; Boron T. III; Mukherjee S.; Christou G.; Pecoraro V. L.; Messinger J.; Yachandra V. K.; Bergmann U.; Yano J. Electronic Structural Changes of Mn in the Oxygen-Evolving Complex of Photosystem II during the Catalytic Cycle. Inorg. Chem. 2013, 52, 5642–5644. 10.1021/ic4005938. 23647530 PMC3683399

[ref83] Chandrasekaran P.; Stieber S. C. E.; Collins T. J.; Que L. Jr.; Neese F.; DeBeer S. Prediction of high-valent iron K-edge absorption spectra by time-dependent Density Functional Theory. Dalton Trans. 2011, 40, 11070–11079. 10.1039/c1dt11331c. 21956429 PMC3242413

[ref84] DeBeer George S.; Petrenko T.; Neese F. Prediction of Iron K-Edge Absorption Spectra Using Time-Dependent Density Functional Theory. J. Phys. Chem. A 2008, 112, 12936–12943. 10.1021/jp803174m. 18698746

[ref85] Rees J. A.; Martin-Diaconescu V.; Kovacs J. A.; DeBeer S. X-ray Absorption and Emission Study of Dioxygen Activation by a Small-Molecule Manganese Complex. Inorg. Chem. 2015, 54, 6410–6422. 10.1021/acs.inorgchem.5b00699. 26061165 PMC4494871

[ref86] Orio M.; Pantazis D. A. Successes, challenges, and opportunities for quantum chemistry in understanding metalloenzymes for solar fuels research. Chem. Commun. 2021, 57, 3952–3974. 10.1039/D1CC00705J. 33885698

[ref87] Drosou M.; Zahariou G.; Pantazis D. A. Orientational Jahn–Teller Isomerism in the Dark-Stable State of Nature’s Water Oxidase. Angew. Chem., Int. Ed. 2021, 60, 13493–13499. 10.1002/anie.202103425. PMC8252073 33830630

[ref88] Corry T. A.; O’Malley P. J. Proton Isomers Rationalize the High- and Low-Spin Forms of the S_2_ State Intermediate in the Water-Oxidizing Reaction of Photosystem II. J. Phys. Chem. Lett. 2019, 10, 5226–5230. 10.1021/acs.jpclett.9b01372. 31429574

[ref89] Pantazis D. A.; Ames W.; Cox N.; Lubitz W.; Neese F. Two Interconvertible Structures that Explain the Spectroscopic Properties of the Oxygen-Evolving Complex of Photosystem II in the S_2_ State. Angew. Chem., Int. Ed. 2012, 51, 9935–9940. 10.1002/anie.201204705. 22907906

[ref90] Lohmiller T.; Krewald V.; Sedoud A.; Rutherford A. W.; Neese F.; Lubitz W.; Pantazis D. A.; Cox N. The First State in the Catalytic Cycle of the Water-Oxidizing Enzyme: Identification of a Water-Derived μ-Hydroxo Bridge. J. Am. Chem. Soc. 2017, 139, 14412–14424. 10.1021/jacs.7b05263. 28921983

[ref91] Saito K.; William Rutherford A.; Ishikita H. Energetics of proton release on the first oxidation step in the water-oxidizing enzyme. Nat. Commun. 2015, 6, 8488 10.1038/ncomms9488. 26442814 PMC4617610

[ref92] Nakamura S.; Noguchi T. Quantum mechanics/molecular mechanics simulation of the ligand vibrations of the water-oxidizing Mn4CaO5 cluster in photosystem II. Proc. Natl. Acad. Sci. U. S. A. 2016, 113, 12727–12732. 10.1073/pnas.1607897113. 27729534 PMC5111704

[ref93] Corry T. A.; O’Malley P. J. Molecular Identification of a High-Spin Deprotonated Intermediate during the S _2_ to S _3_ Transition of Nature’s Water-Oxidizing Complex. J. Am. Chem. Soc. 2020, 142, 10240–10243. 10.1021/jacs.0c01351. 32431144

[ref94] Zimmermann J. L.; Rutherford A. W. Electron paramagnetic resonance properties of the S_2_ state of the oxygen-evolving complex of photosystem II. Biochemistry 1986, 25, 4609–4615. 10.1021/bi00364a023.

[ref95] Yamamoto M.; Nakamura S.; Noguchi T. Protonation structure of the photosynthetic water oxidizing complex in the S0 state as revealed by normal mode analysis using quantum mechanics/molecular mechanics calculations. Phys. Chem. Chem. Phys. 2020, 22, 24213–24225. 10.1039/D0CP04079G. 33084674

[ref96] Leto D. F.; Jackson T. A. Mn K-Edge X-ray Absorption Studies of Oxo- and Hydroxo-manganese(IV) Complexes: Experimental and Theoretical Insights into Pre-Edge Properties. Inorg. Chem. 2014, 53, 6179–6194. 10.1021/ic5006902. 24901026 PMC4066903

[ref97] Bhowmick A.; Hussein R.; Bogacz I.; Simon P. S.; Ibrahim M.; Chatterjee R.; Doyle M. D.; Cheah M. H.; Fransson T.; Chernev P.; Kim I. S.; Makita H.; Dasgupta M.; Kaminsky C. J.; Zhang M.; Gatcke J.; Haupt S.; Nangca I. I.; Keable S. M.; Aydin A. O.; Tono K.; Owada S.; Gee L. B.; Fuller F. D.; Batyuk A.; Alonso-Mori R.; Holton J. M.; Paley D. W.; Moriarty N. W.; Mamedov F.; Adams P. D.; Brewster A. S.; Dobbek H.; Sauter N. K.; Bergmann U.; Zouni A.; Messinger J.; Kern J.; Yano J.; Yachandra V. K. Structural evidence for intermediates during O(2) formation in photosystem II. Nature 2023, 617, 629–636. 10.1038/s41586-023-06038-z. 37138085 PMC10191843

[ref98] Greife P.; Schonborn M.; Capone M.; Assuncao R.; Narzi D.; Guidoni L.; Dau H. The electron-proton bottleneck of photosynthetic oxygen evolution. Nature 2023, 617, 623–628. 10.1038/s41586-023-06008-5. 37138082 PMC10191853

