# Supplementary material for: Nature of S-States in the Oxygen-Evolving Complex Resolved by High-Energy Resolution Fluorescence Detected X-ray Absorption Spectroscopy
Source: J Am Chem Soc. 2023 Nov 16;145(47):25579–94. doi: 10.1021/jacs.3c06046 (PMC10690802; doi:10.1021/jacs.3c06046)
Supplement: Supplementary file 1 — ja3c06046_si_001.pdf [file ja3c06046_si_001.pdf]

SUPPORTING INFORMATION

for

Nature of S-States in the Oxygen-Evolving Complex Resolved by High-Energy Resolution Fluorescence Detected X-ray Absorption Spectroscopy

Maria Chrysina, Maria Drosou, Rebeca G. Castillo, Michael Reus, Frank Neese, Vera Krewald, Dimitrios A. Pantazis,* Serena DeBeer*

Contents:

1. Quantification of the S-states of Samples Using EPR Spectroscopy 2
2. S_2 and S_3 Heterogeneity 5
   Heterogeneity of the S_2 State 5
   Heterogeneity of the S_3 State 5
3. Damage Studies 7
4. Experimental Error Evaluation 8
5. Quantum Chemical Cluster Model of the OEC 9
6. Derivatives of the Pure S-state Spectra 10
   First Derivatives of the Pure S-state Spectra 10
   Second Derivatives of the Pure S-state Spectra 11
7. Comparison with Previous Data 12
8. Fitting of the Pre-Edge Peaks with Voigt Curves 14
9. S_i–S_{i-1} Differences Error 16
10. Calculated Mn XAS Pre-Edge Spectra for All Computational Models 17
11. Assignment of the Calculated Pre-Edge XAS Spectra 20
12. Analysis of Individual Mn Ion Contributions in the S_3 State Pre-edge XAS Spectra 26
13. Example of XAS Orca 5 Input 27
References 28

1. Quantification of the S-states of Samples Using EPR Spectroscopy

The progression of PS II samples to the next S-transition upon illumination is not perfect, thus quantification of the population of PS II centers of the sample in each S-state is essential. EPR spectroscopy was employed following the protocol of Messinger et al.[1] The multiline signal of S_2 state at $g \approx 2$ was measured in all samples in order to monitor the evolution of the S_2 population after each visible light flash. In Figure S1, representative spectra of a series of samples after 1–5 laser flashes are presented; they are subtraction spectra minus the S_1 background. In the S_1 state there was no sign of multiline spectrum that would be originated from any residual S_2 state caused by the preflash. All spectra were normalized to the PS II content with respect to the g_x signal of cytochrome c_{550} of the extrinsic subunit of PS II at 2300 G. The multiline intensity for each sample was estimated by the average of the peak-to-peak intensity of the lines indicated in Figure S1a. The intensity of the multiline after 1 flash, i.e. in the S_2 state, was assumed to be 100% and the multiline after 2–5 flashes was calculated as a percentage of the intensity of S_2. The average of the multiline intensity of all samples after each number of flashes is presented in Table 1 and in Figure S1b as blue circles.

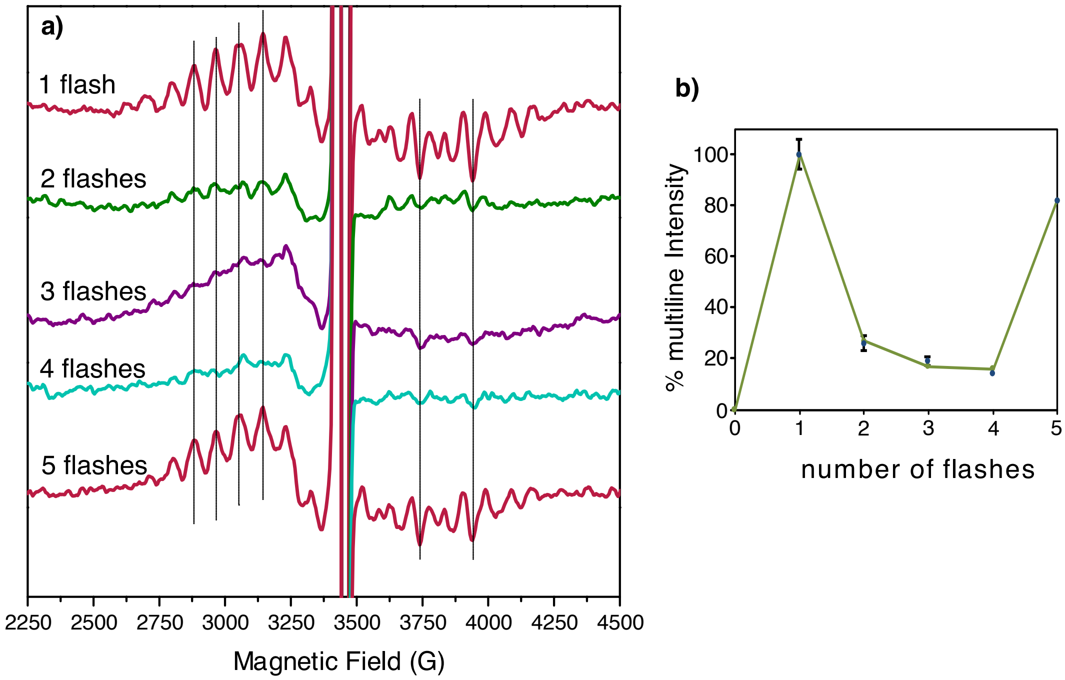

Figure S1. (a) Representative EPR spectra after 1–5 flashes. All spectra are subtracted minus the dark S_1 state in order to eliminate the g_y component from cytc_{550} that interferes with the multiline signal. The peaks used for measuring the multiline intensity after each flash are marked by dashed lines. EPR parameters: modulation frequency: 100 kHz, modulation amplitude: 7.5 G, microwave frequency: 9.6 GHz, microwave power: 8 mW, sweep time: 84 s, sweep field: 5000 G, average of 4 scans, temperature: 10 K. **(b)** Experimental multiline signal intensity after 1–5 flashes (blue circles) in comparison with the calculated multiline intensity fitted with parameters: miss factor = 0.06 and deficiency at S_2 = 0.15. Blue circles are the average of multiline intensity of all samples; the standard deviation is marked by bars.

In order to calculate the S-state content of samples after each flash a model including misses, double hits, initial S_2 population and/or blocking at S_2 was used. Different combinations of the above parameters were tested in order to fit the multiline intensity calculated using this model with the experimental; residuals of the experimental and calculated multiline intensity were used as an estimate of the error of the fitting. Some representative trials are presented in Table 1. First, a miss factor of 10% was used; higher miss factors were also tried but increased the error. It is noted that the same miss factor for all S-transitions was used; deviations that may exist are out of the resolving capability of the HERFD experiment. Consideration of double hits did not improve the fitting. Double hits are not expected anyway when laser is used for illumination of the samples.

After 3 and 4 flashes there is 20% of multiline intensity that cannot be explained only by misses (see Table 1). Consideration of a population of PS II centers blocked at S_2 state that do not progress further upon illumination improved the fitting. In these centers the acceptor side of PS II is deficient and cannot accept electrons from the OEC, to progress beyond the S_2 state. A value of 5% that is similar with that in Messinger et al. was initially used. Consideration of an initial S_2 population in dark-adapted samples led to a small improvement, but on the way smaller miss factor and higher population of centers blocked at S_2 improved the fitting more significantly without the initial S_2 contribution. No multiline signal could be observed in S_1 spectra (not shown), consistent with the fact that no initial S_2 population was used. Finally, the best fitting between the experimental and the calculated multiline intensity was achieved by using a miss factor of 6% and 15% blockage at S_2. In a separate experiment, a singly-flashed sample was illuminated with continuous visible light illumination at -80 °C in order to create the maximum S_2 population and no increase of the S_2 multiline signal was observed. This indicates that we have indeed ~ 100% S_2 after the first flash. The absense of delay in S-state advancement during the $S_1 \rightarrow S_2$ transition confirms that after dark adaptation the samples are synchronized in the S_1 state with 100% oxidized Y_D. The blockage at S_2 state is too high. Exogenous electron acceptor has been added but it may not be integrated well in all PS II centers. To the end, the population of each S-state after each laser flash is presented in Table 2. After "0, 1, 2, 3 flashes" there is 100% S_1, 94% S_2, 74% S_3, and 70% S_0 respectively.

Table S1. Calculated multiline intensity in comparison with the experimental intensity of the multiline signal of the S_2 state using different combinations of parameters. All parameters are given as percentages (%).

									experimental multiline intensity
	misses	10	10	10	10	10	7	6	
	double hits	0	5	0	0	0	0	0	
	impair at S_2	0	0	5	5	10	10	15	
	initial S_2	0	0	0	5	5	5	0	
calculated multiline intensity	1 flash	100	100	100	100	100	100	100	100
	2 flashes	20	20	25	25	30	24	27	26
	3 flashes	3	4	9	8	14	12	17	19
	4 flashes	0	15	6	10	15	15	16	14
	5 flashes	66	60	68	69	71	79	82	82
	Error[1]	744	771	384	296	163	63	9	

[1] The error of each fitting was calculated as the sum of residuals between calculated and experimental multiline intensity, as in Messinger et al.[1]

Table S2. Calculated S-state content after a given number of flashes.

# flashes	S_1	S_2	S_3	S_0
0	**100**			
1	6	**94**		
2	1	25	**74**	
3	0	16	14	**70**
4	65	15	2	18
5	20	77	0	3

2. S_2 and S_3 Heterogeneity

Heterogeneity of the S_2 State

It was mentioned in the main text that we avoided using glycerol in the samples in order to achieve a homogeneous S_3 that has been already characterized by EPR, hyperfine spectroscopy and DFT.[2] This choice has the drawback that a minority of centers of S_2 state represents the high spin form. In order to quantify this minor S_2 population of our samples we compared the present S_2 with methanol treated S_2 (Figure 2). Methanol converts the minor component of S_2 (high spin) to the major component (low spin) that is represented by the multiline signal. In the methanol treated sample, an increase in the intensity of the S_2 multiline signal is observed by 20%, which implies that the untreated sample contains 20% of the minor high-spin component of S_2. Thus, the S_2 state of this study represents the low-spin form in 80% of centers.

Heterogeneity of the S_3 State

W-band EPR/EDNMR and DFT revealed an open cubane structure of the S_3 state with four 6-coordinate Mn^{IV} in the thermophilic cyanobacterium *T. vestitus*; a new water that is not present in S_2 is bound to Mn1.[2] The EPR spectrum is shown in green in Figure S2b. Three perturbed configurations of this motif were observed in glycerol treated PS II from cyanobacterium *Synechocystis* sp by 130 GHz EPR.[3] A form of the S_3 state with a Mn ion to represent highly

anisotropic hyperfine couplings was observed by W-band EPR/EDNMR in methanol or glycerol treated or Sr-substituted samples from *T. vestitus*.[4] The aforementioned S_3 forms represent a total spin of $S = 3$. A high spin S_3 form ($S = 6$) was observed by Q-band EPR spectroscopy in methanol treated PS II from spinach and also as a major component in untreated PS II,[5] as first predicted by quantum chemical studies.[6]

In Figure S2b representative spectra of the two configurations of the S_3 state in *T. vestitus* are presented. The first observed high-field EPR signal of the S_3 ("narrow") is shown in green. Upon addition of glycerol or methanol in the samples, a "wide" component is observed (marked with the red arrow) together with the "narrow" signal. The populations of each component depend on the percentage of methanol of glycerol added. We used glycerol-free samples that represent only the "narrow" S_3 (green in Figure S2b).

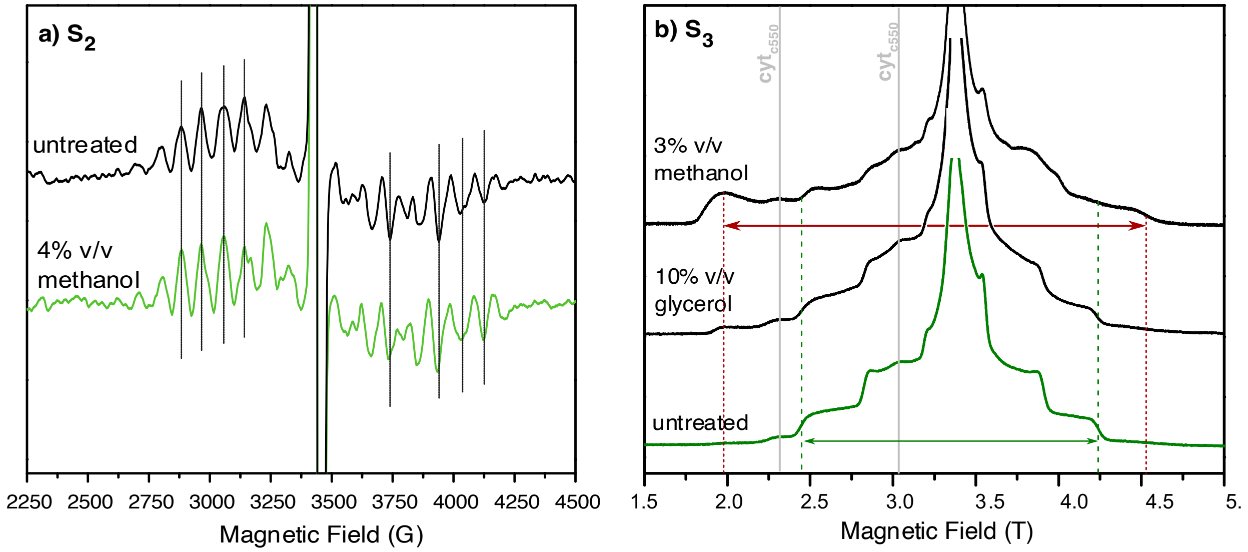

Figure S2. Inhomogeneity of the S_2 (a) and S_3 (b) states as indicated by X-band and W-band EPR spectroscopy respectively. The green spectra in both figures are the homogeneous S_2 and S_3 configurations, while the black represent mixtures (see text). In the samples used herein the S_2 is inhomogeneous (black in panel a), while the S_3 is homogeneous (green in panel b). **(a)** Comparison of the S_2 state in untreated and methanol-treated PS II. The 20% increase of the multiline intensity upon addition of methanol indicates that 20% of the population in the untreated S_2 represents a high spin form, not observable by X-band. EPR parameters as in Figure S1. **(b)** W-band ESE-detected field-swept spectra of the untreated S_3 (green), S_3 with 10% glycerol and 3% methanol (black). Cytochrome signals are marked with grey lines. The untreated S_3 signal (narrow) is marked by a green arrow while the recently revealed configuration of S_3 (wide) by a red arrow. Panel S2b adapted from Chrysina et al. *PNAS* **2019**, 116 (34), 16841-16846. Copyright 2019 National Academy of Sciences

3. Damage Studies

Before starting the actual measurement of each sample (no flash, 1, 2, 3 flashes), damage studies were performed (described in methods section). Here, some representative spectra are presented.

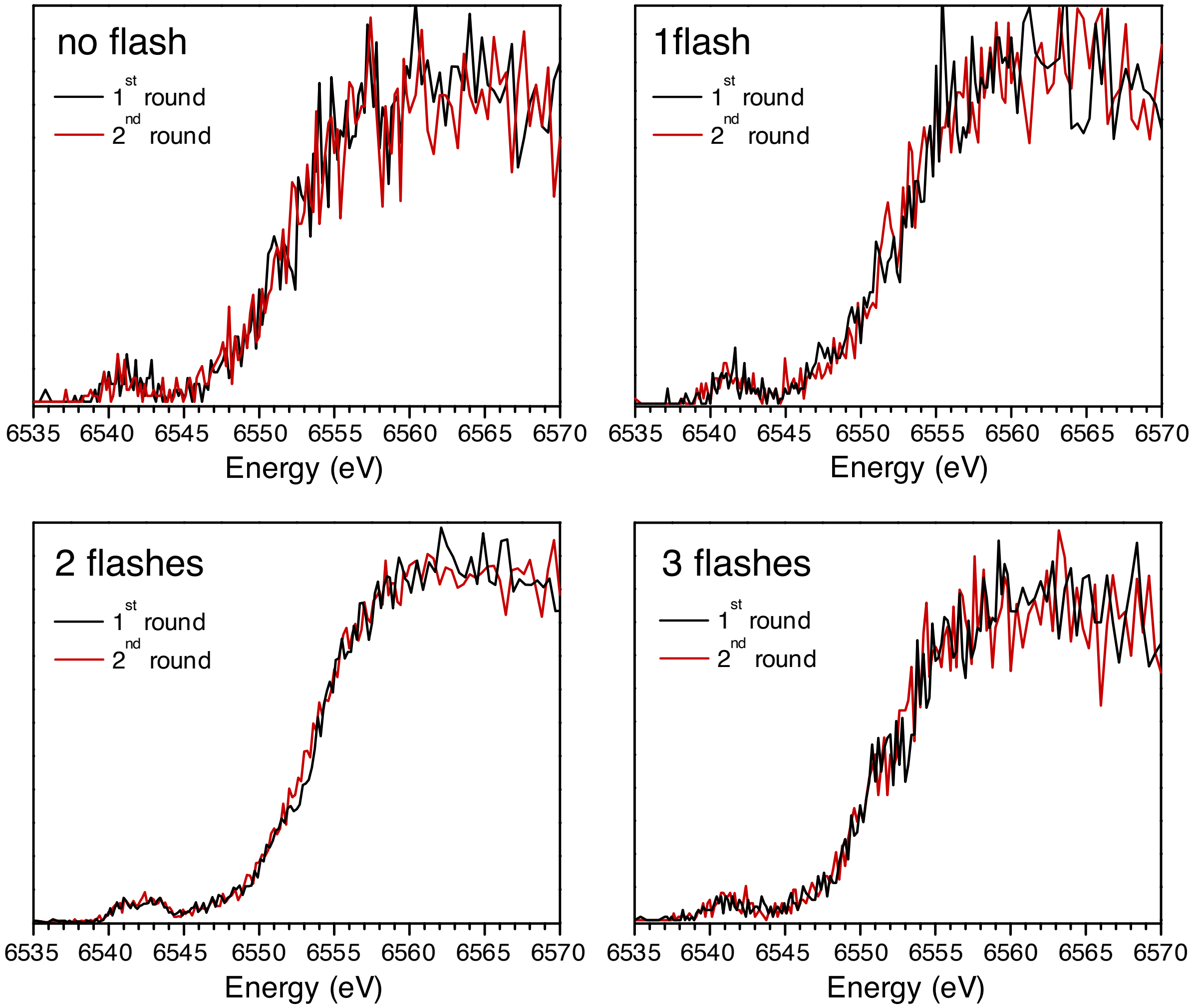

Figure S3. Damage test in 0, 1, 2, 3 flashed samples: 2 rounds of measurement were performed on the same spots. In all cases flux was attenuated to 6%. For 0 and 1 flashed sample (S_1 and S_2 samples): scan time was 58 s, average of three different spots was used. No damage was observed in 116 s. In 2 and 3 flashed samples, each scan is 38 s. No damage was observed in 76 s. For the 2-flashed sample (mainly S_3 state sample) the average of 37 spots was tested for damage and for the 3-flashed sample (mainly S_0 state sample), the average of 8 spots.

4. Experimental Error Evaluation

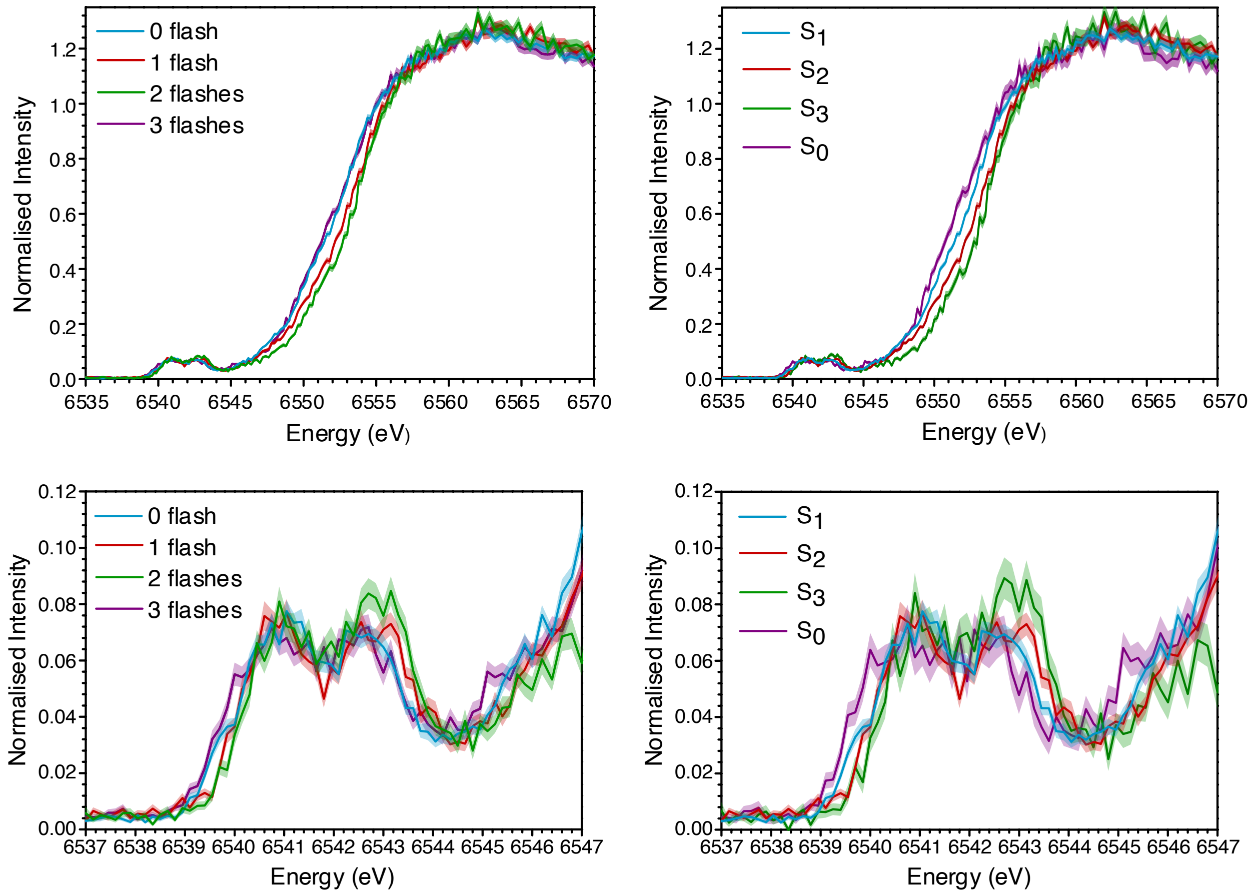

Figure S4. Standard error of the 0, 1, 2, 3 flashed spectra (left) and of the pure S_1, S_2, S_3, S_0 spectra (right) is represented as a shaded area. Standard error was calculated as described in Material and Methods.

5. Quantum Chemical Cluster Model of the OEC

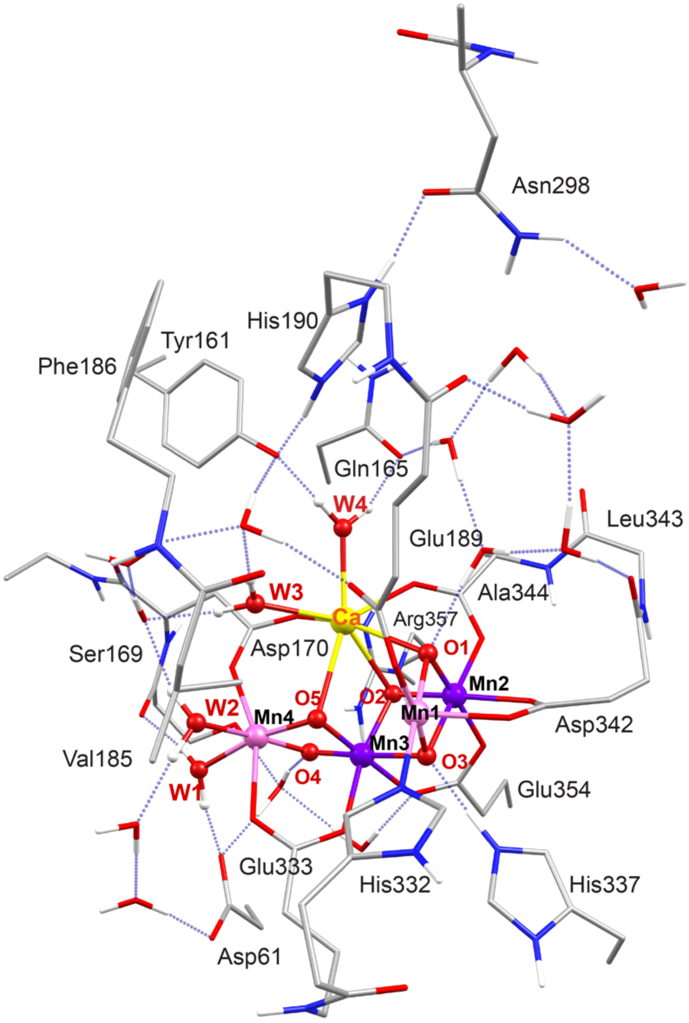

Figure S5. QM Model of the S_1 state. H atoms attached to carbons are omitted for clarity.

6. Derivatives of the Pure S-state Spectra

First Derivatives of the Pure S-state Spectra

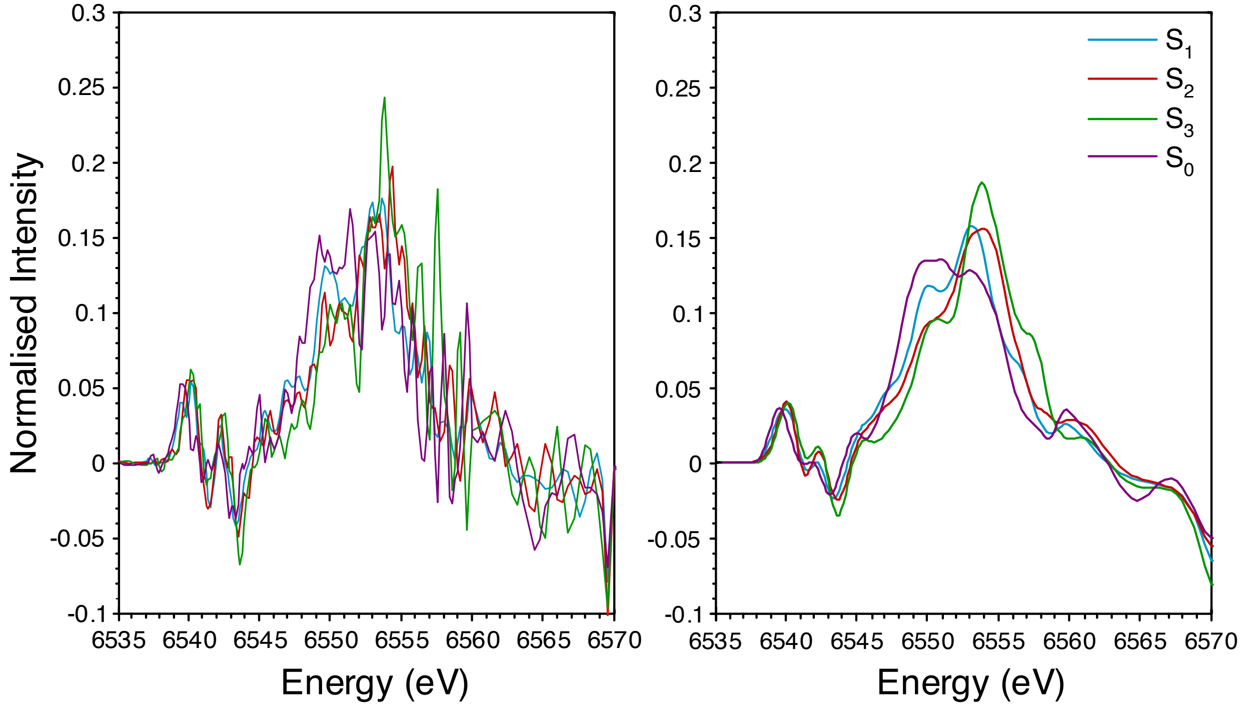

Figure S6. First derivative (with no smoothing at left and 41 point smoothing at right) of the XANES spectra (5 point smoothed). The XANES spectra were 5-point smoothed, the first derivative was calculated and the first derivative was 41-point smoothed.

Second Derivatives of the Pure S-state Spectra

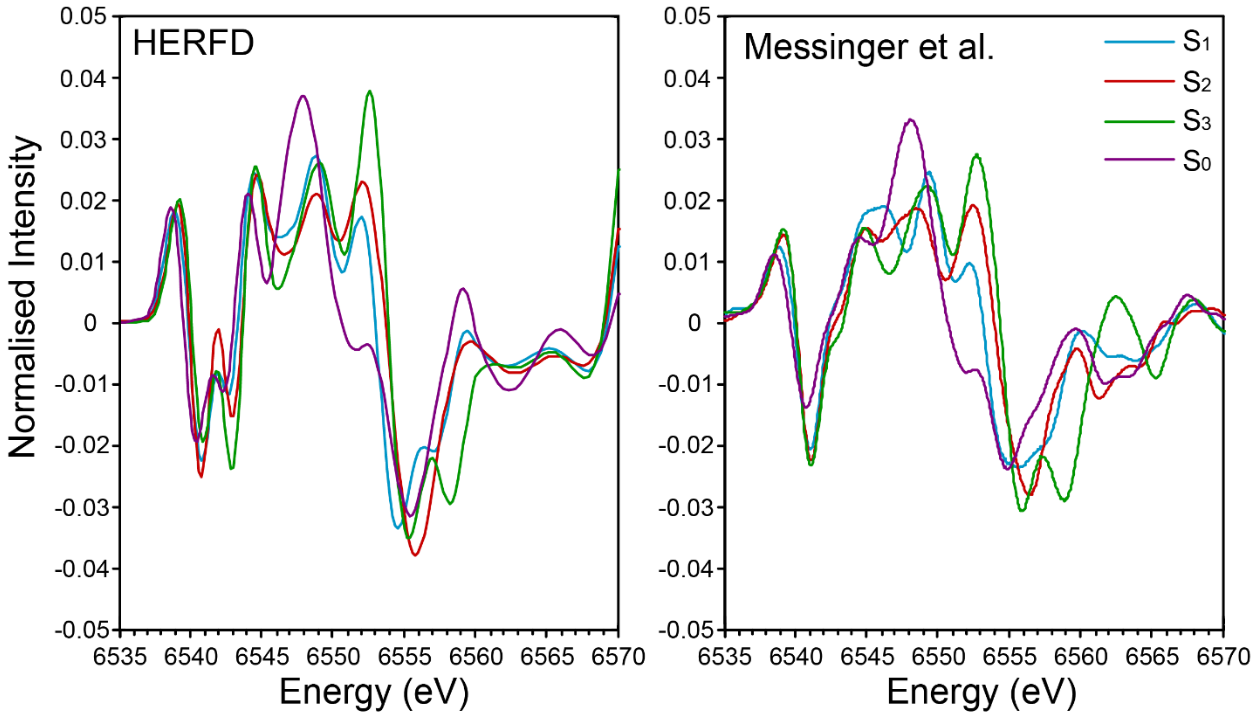

Figure S7. Second derivative of the HERFD data (left) in comparison to Messinger et al.[1] second derivative (right).

7. Comparison with Previous Data

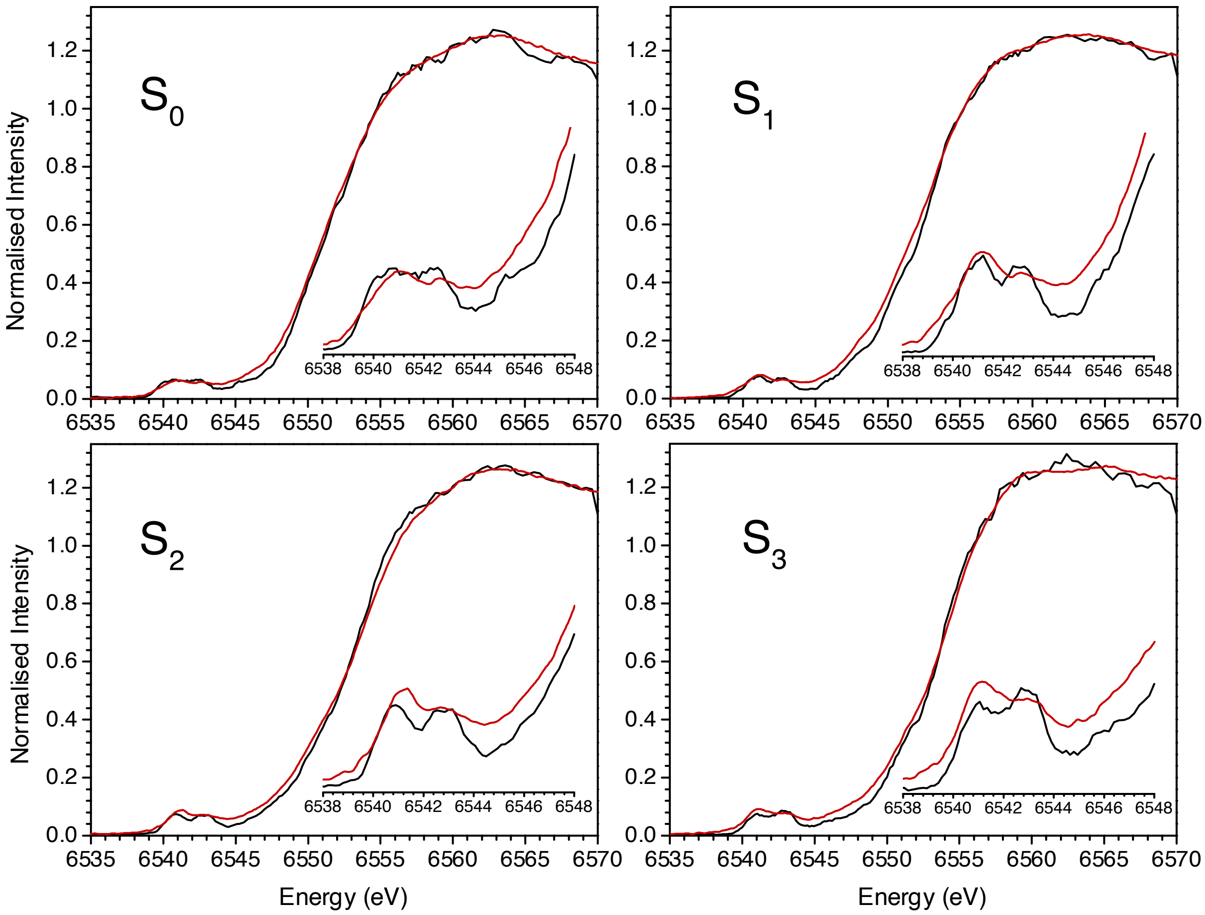

Figure S8. HERFD spectra of this work (black) in comparison with data reported from Messinger et al.[1] (red). Adapted in part from Messinger at al. J. Am. Chem. Soc. 2001, 123, 7804-7820. Copyright 2001 American Chemical Society.

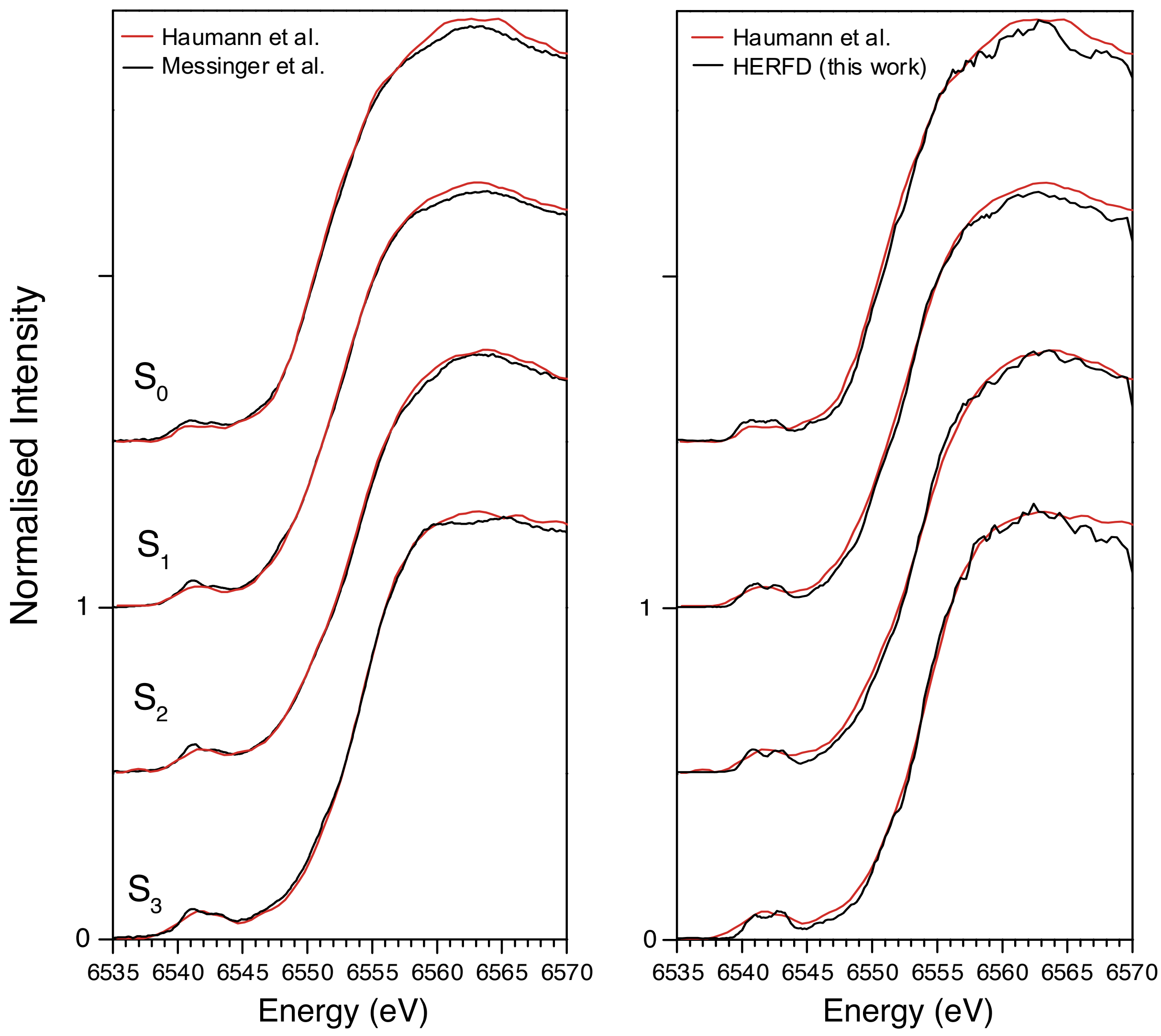

Figure S9. Left: Comparison between XAS spectra of Haumann et al.[7] (in red) and Messinger et al.[1] (in black) Right: Comparison between XAS spectra of Haumann et al.[7] (in red) and HERFD spectra of this work (in black). Adapted in part from Messinger at al. *J. Am. Chem. Soc.* **2001**, 123, 7804-7820 (Copyright 2001 American Chemical Society) and Haumann et al *Biochemistry* **2005**, *44*, 1894-1908 (Copyright 2005 American Chemical Society).

Haumann et al.[7] data are in spinach, 1M betaine as a cryoprotectant and 10%v/v glycerol to maximize the multiline S_2 signal. No g = 4.1 was reported to be observed, thus S_2 is assumed to be 100% in the low-spin form. Partially dehydrated samples. Messinger et al.[1] experiments are in spinach, 30% glycerol. There are no high field EPR data in spinach S_3 with glycerol at the moment.

8. Fitting of the Pre-Edge Peaks with Voigt Curves

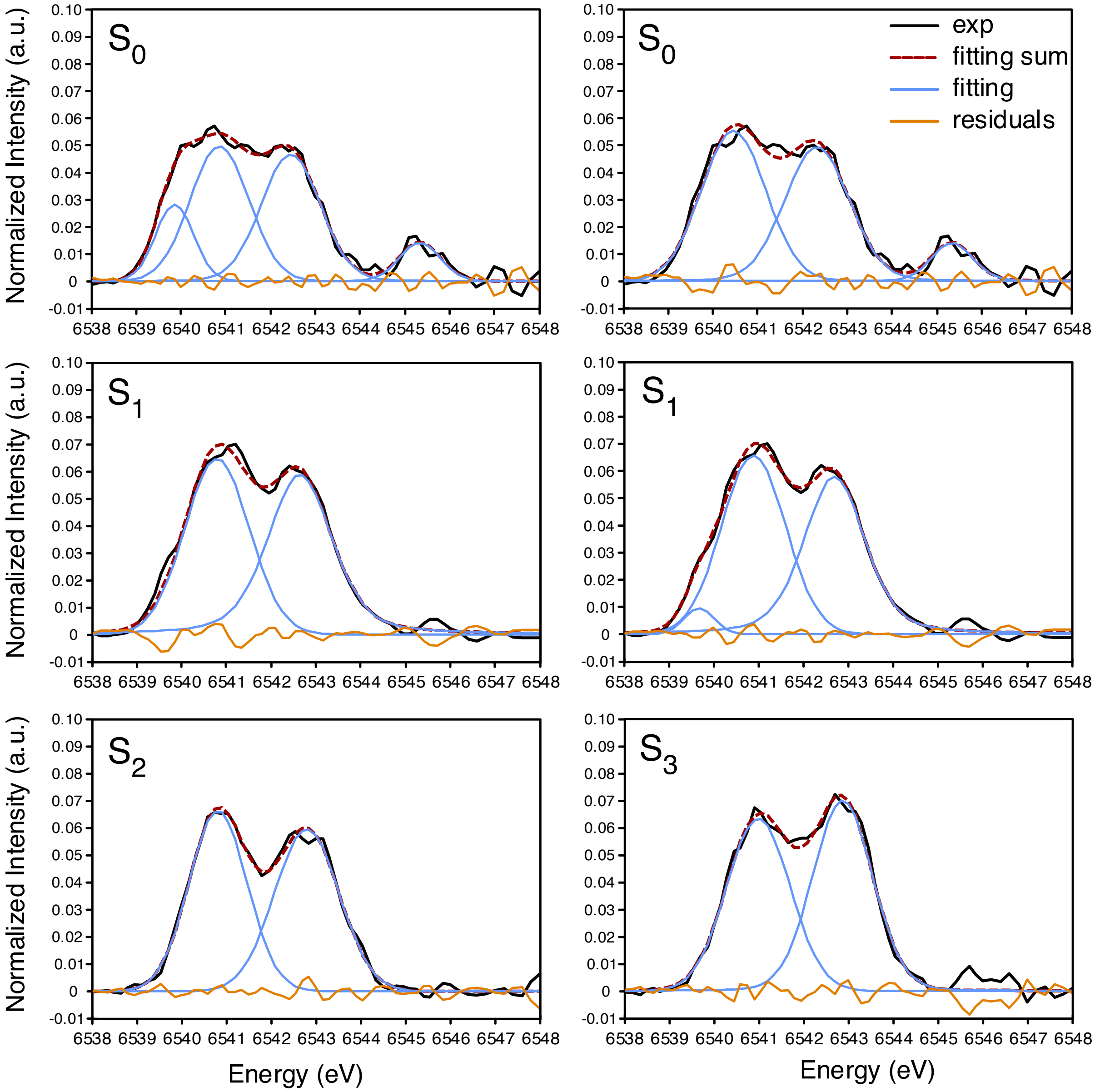

Figure S10. Fitting of the pre-edge experimental peaks (black) with Voigt curves (red). The individual curves used for the fitting are presented in blue and the residuals in orange. Fitted parameters are listed in Table S3.

Table S3. Parameters of the fitting of the pre-edge region.

	Amplitude	Energy (eV)	Gauss FWHM	Lorentz FWHM	Amplitude	Energy (eV)	Gauss FWHM	Lorentz FWHM
S_0								
Fitting 1 (error = 280.2 10^{-6})					Fitting 2 (error = 455.4 10^{-6})			
peak 1	0.030	6539.864	1.007	0.000	0.100	6540.461	1.700	0.000
peak 2	0.082	6540.866	1.549	0.000	0.092	6542.326	1.700	0.100
peak 3	0.080	6542.455	1.549	0.100	0.017	6545.338	1.097	0.100
peak 4	0.017	6545.336	1.085	0.084				
S_1								
Fitting 1 (error = 324.5 10^{-6})					Fitting 2 (error = 190.2 10^{-6})			
					0.008	6539.681	0.806	0.000
peak 1	0.119	6540.799	1.700	0.027	0.118	6540.896	1.692	0.000
peak 2	0.124	6542.651	1.683	0.463	0.115	6542.703	1.607	0.435
S_2 (error = 226.8 10^{-6})								
peak 1	0.108	6540.816	1.527	0.001				
peak 2	0.108	6542.802	1.700	0.000				
S_3 (error = 449.1 10^{-6})								
peak 1	0.113	6540.994	1.670	0.000				
peak 2	0.122	6542.878	1.573	0.100				

Two options are presented for the fitting of S_0 and S_1 states with Voigt curves. For S_0, the error is lower for fitting 3 peaks in the range 6538-6545 eV rather than 2 peaks (intensity of residuals lower). In the case of S_1, the error is lower for fitting 3 peaks in this range; the one small intensity peak fits a shoulder at 6539.5 eV. The error of the fitting for the S_3 state is high but mainly because of the range > 6545 eV (higher intensity of residuals).

9. S_i–S_{i-1} Differences Error

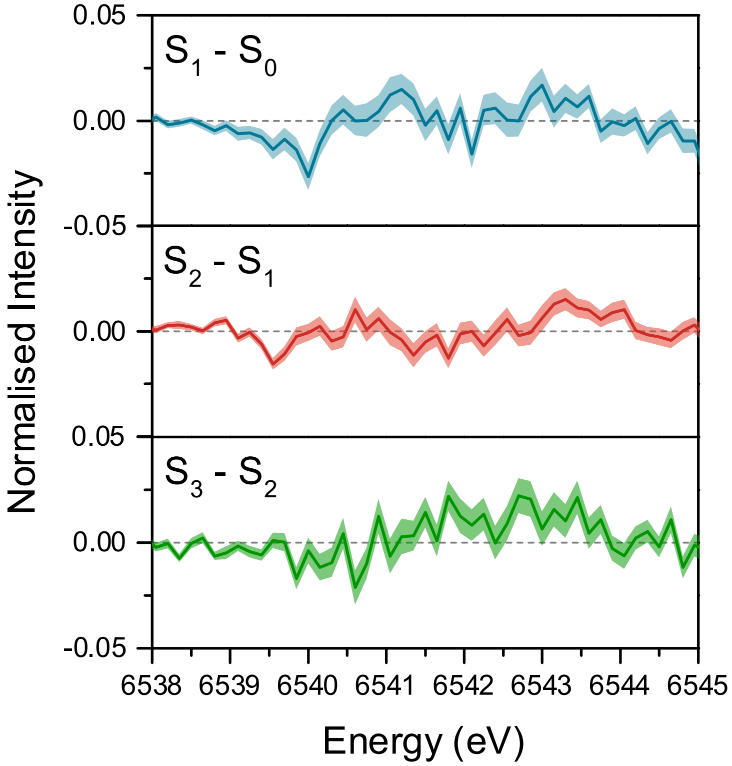

Figure S11. Standard error of the S_i–S_{i-1} differences.

10. Calculated Mn XAS Pre-Edge Spectra for All Computational Models

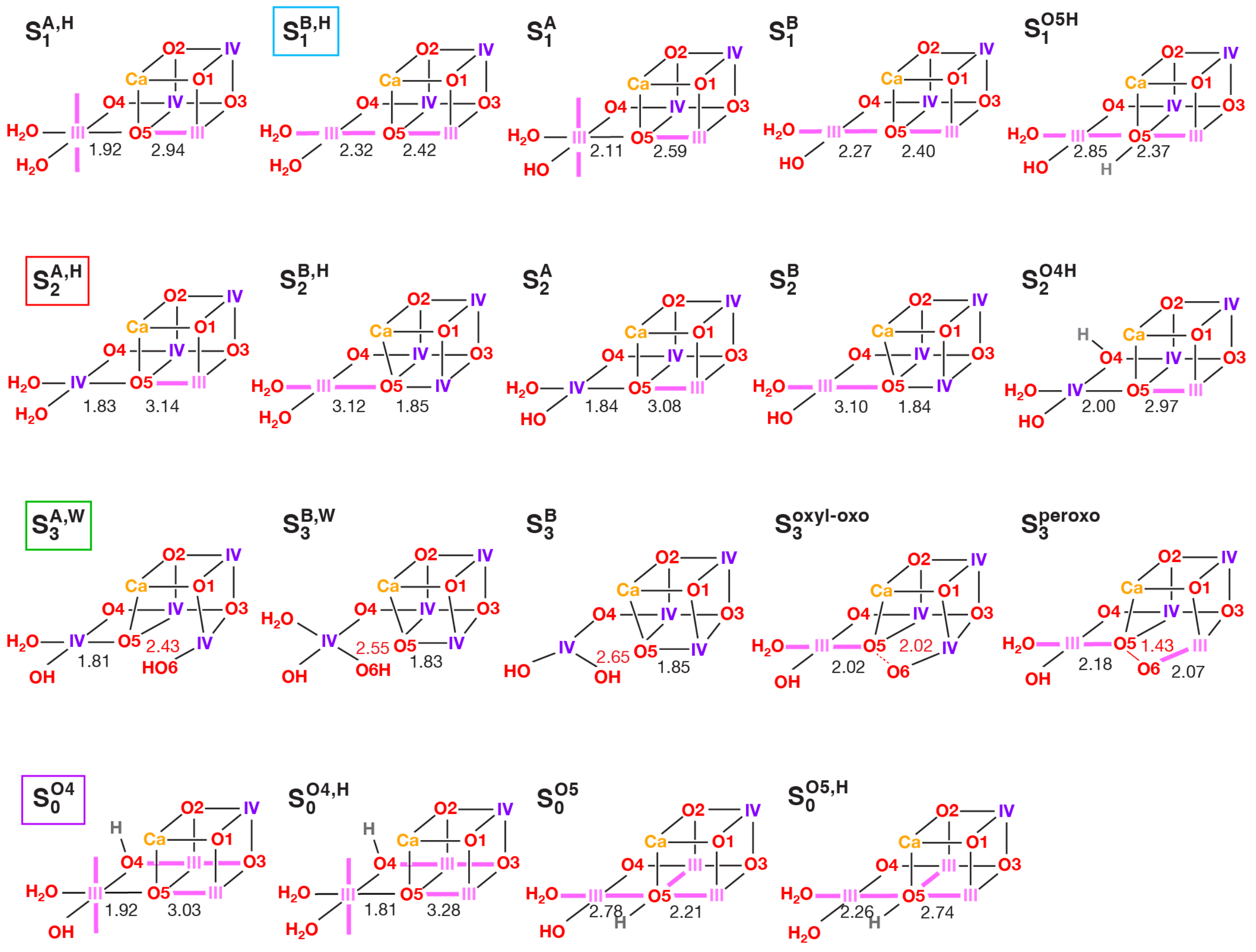

Figure S12. Schematic depiction of the inorganic cores of all S_0–S_3 models examined in this work, with the Mn oxidation states indicated in purple for Mn(IV) and pink for Mn(III), and the Jahn–Teller elongation axis of each Mn(III) ion shown with thicker pink lines. Selected optimized distances (in Å, Mn1/Mn4–O5 in black and O5–O6/W2 in red) are indicated to facilitate comparisons. Complete coordinates of all models are provided in full as Supporting Information.

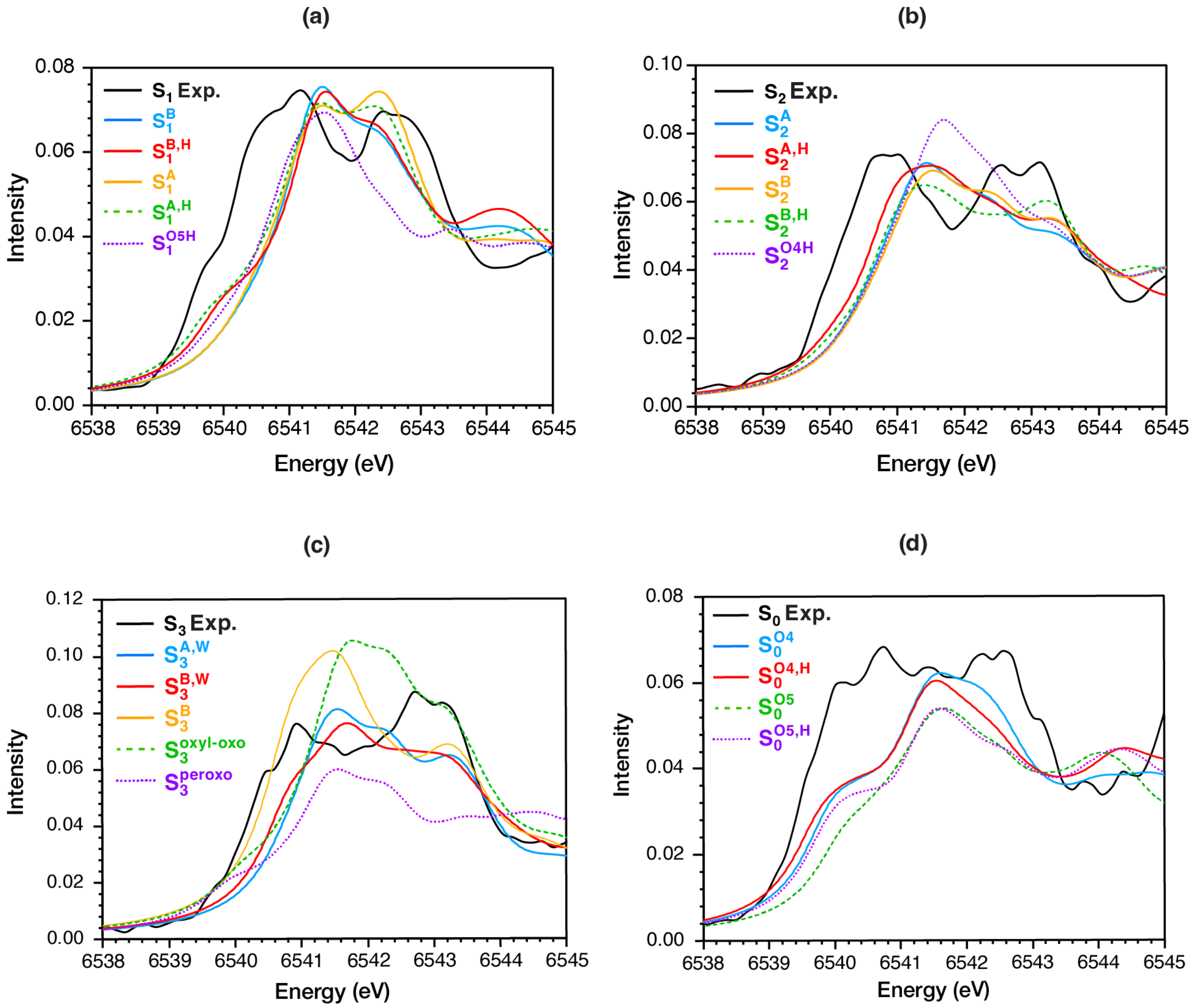

Figure S13. Calculated XAS spectra of all models for each state shown in different colors compared to experiment (black curves). A 1.1 eV broadening and a constant shift of 36.3 eV have been applied to the calculated spectra.

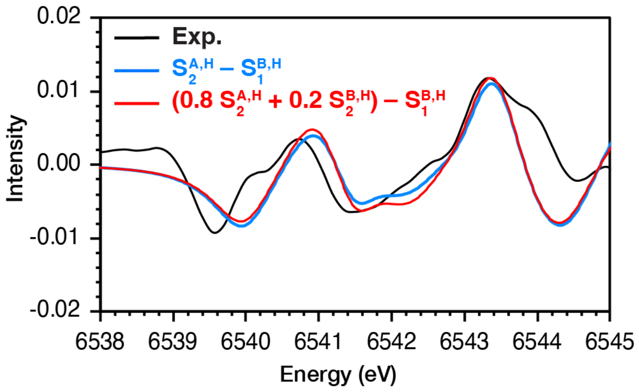

Figure S14. Comparison of experimental S_2–S_1 data (black line) with the calculated S_2–S_1 difference spectra for models $\mathbf{S_1^{B,H}}$ and $\mathbf{S_2^{A,H}}$ (blue line) and with the calculated S_2–S_1 difference spectra for model $\mathbf{S_1^{B,H}}$ and a mixture of 80% $\mathbf{S_2^{A,H}}$ and 20% $\mathbf{S_2^{B,H}}$ (red line). The effect of 20% $\mathbf{S_2^{B,H}}$ admixture on the difference spectra is negligible and both S_2–S_1 difference spectra reproduce experiment.

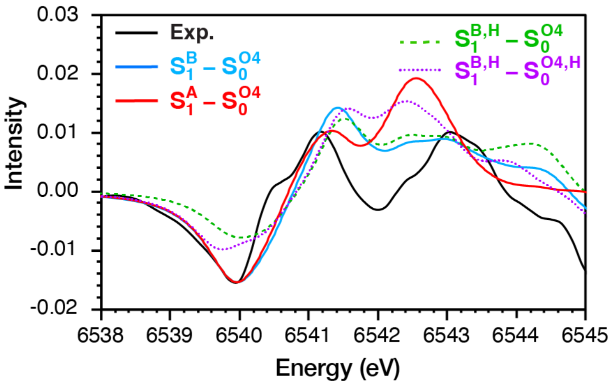

Figure S15. Experimental and calculated S_1–S_0 spectral differences for different of S_0 and S_1 isomers.

11. Assignment of the Calculated Pre-Edge XAS Spectra

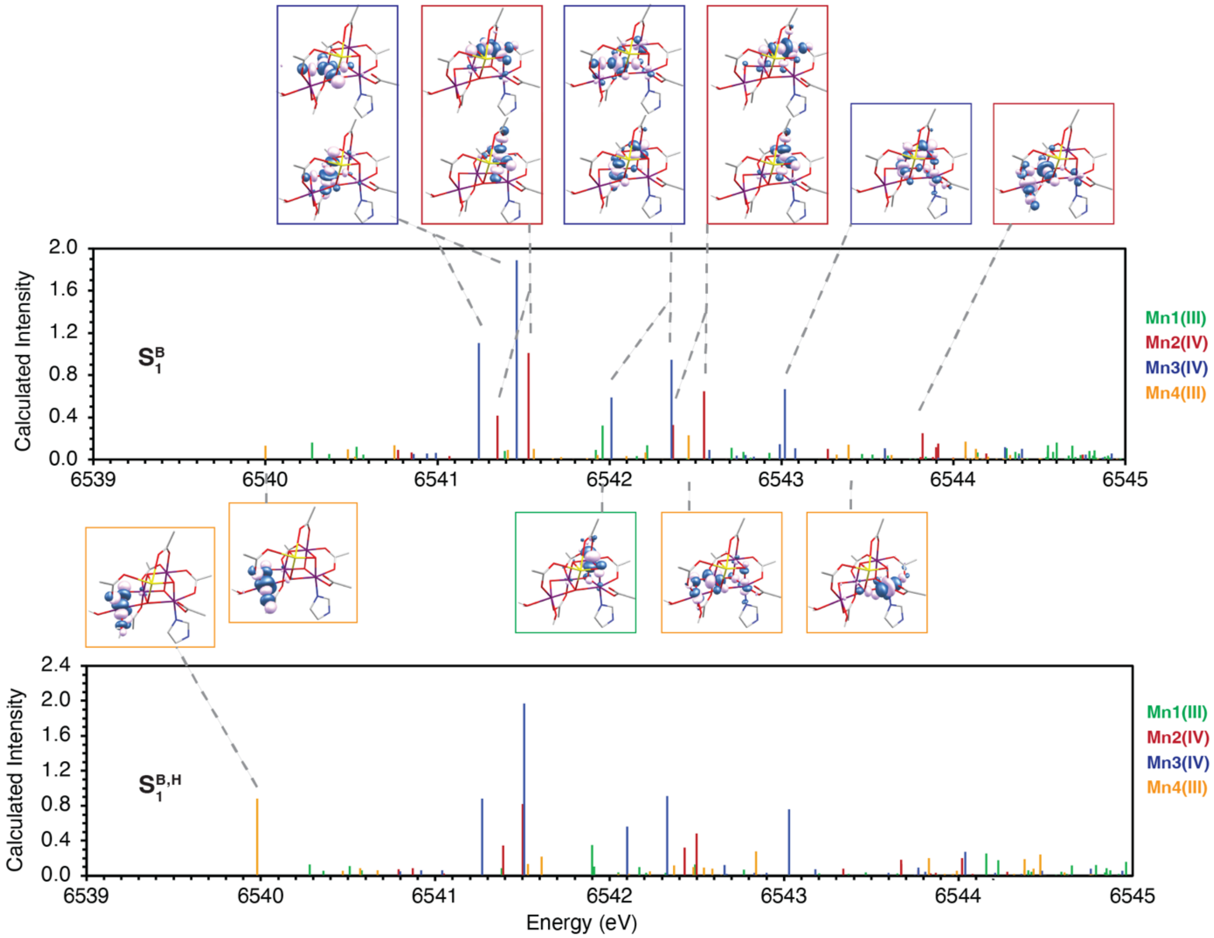

Figure S16. Assignment of the calculated pre-edge XAS spectrum based on the NTOs associated with the transitions for models $\mathbf{S_1^B}$ and $\mathbf{S_1^{B,H}}$. Assignment of transitions for $\mathbf{S_1^{B,H}}$ is similar to that of $\mathbf{S_1^B}$. The local Mn4 1s → 3d transition at ~6540 eV is more intense in $\mathbf{S_1^{B,H}}$.

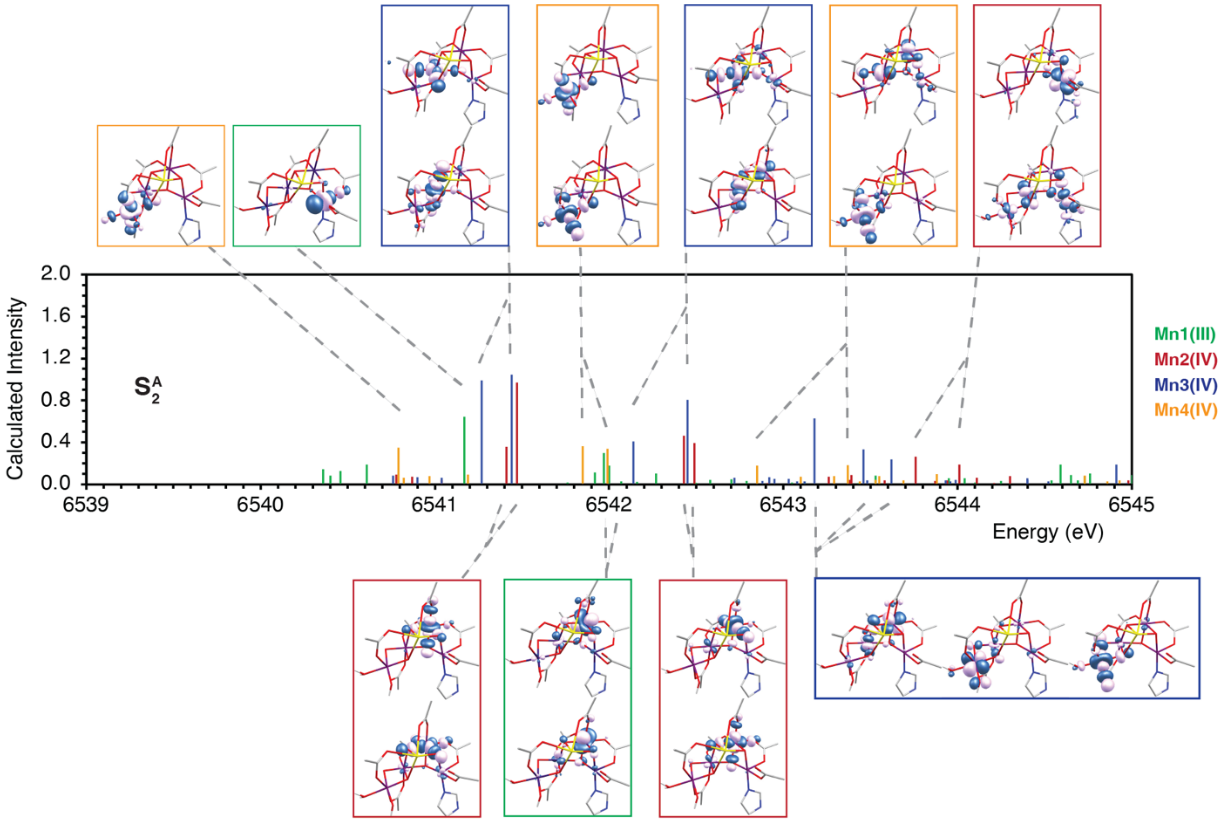

Figure S17. Assignment of the calculated pre-edge XAS spectrum based on the NTOs associated with the transitions for model $\mathbf{S_2^A}$.

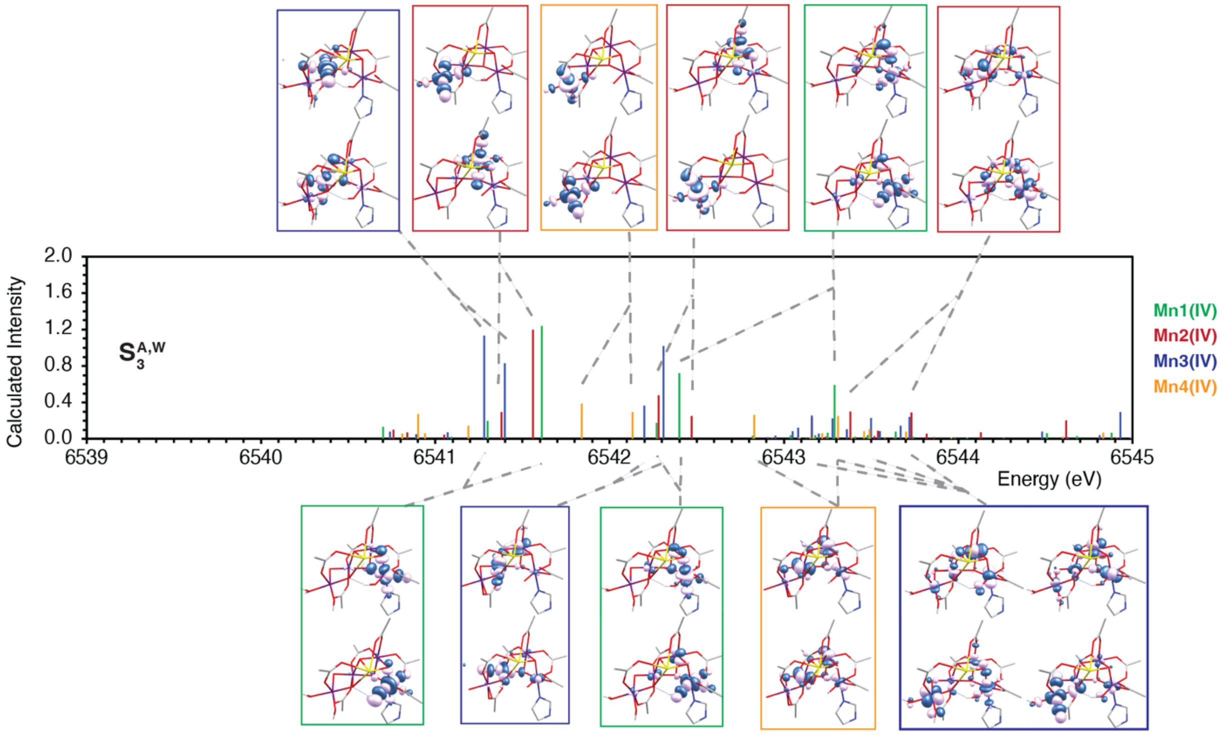

Figure S18. Assignment of the calculated pre-edge XAS spectrum based on the NTOs associated with the transitions for model $\mathbf{S_3^{A,W}}$.

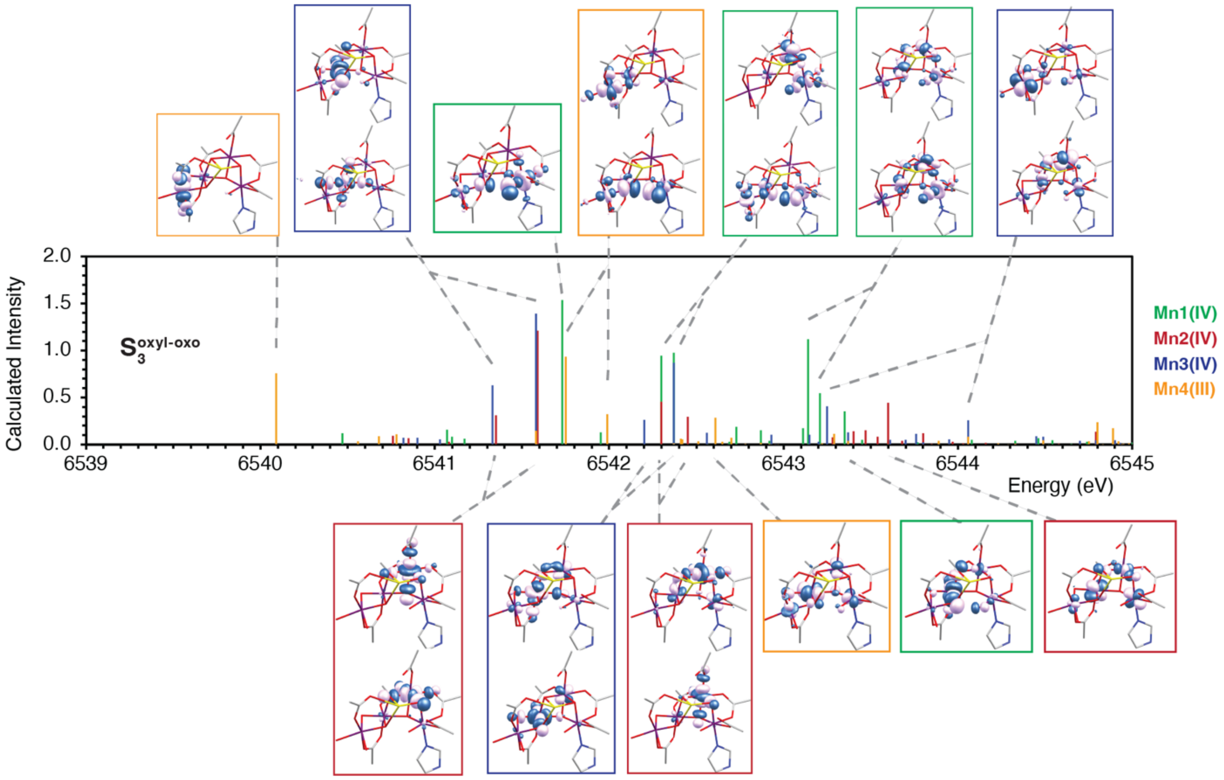

Figure S19. Assignment of the calculated pre-edge XAS spectrum based on the NTOs associated with the transitions for model $\mathbf{S_3^{oxyl-oxo}}$.

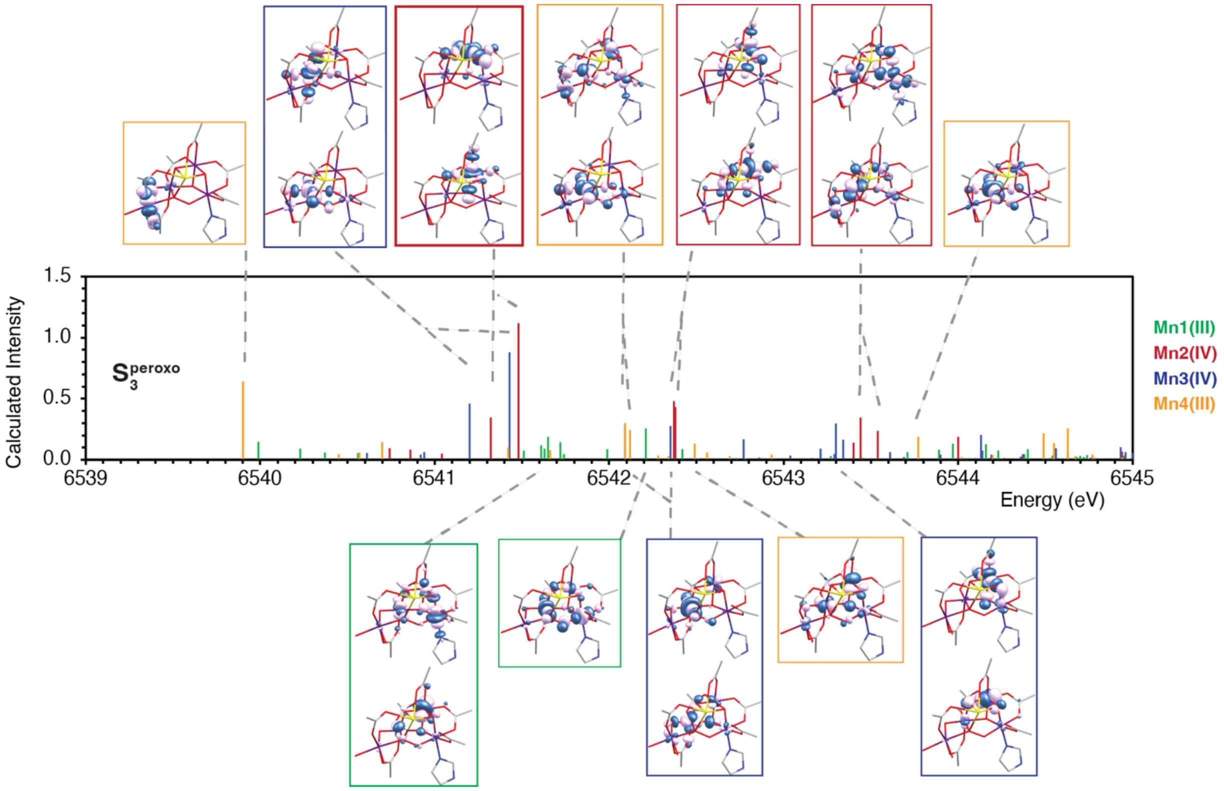

Figure S20. Assignment of the calculated pre-edge XAS spectrum based on the NTOs associated with the transitions for model $\mathbf{S_3^{peroxo}}$.

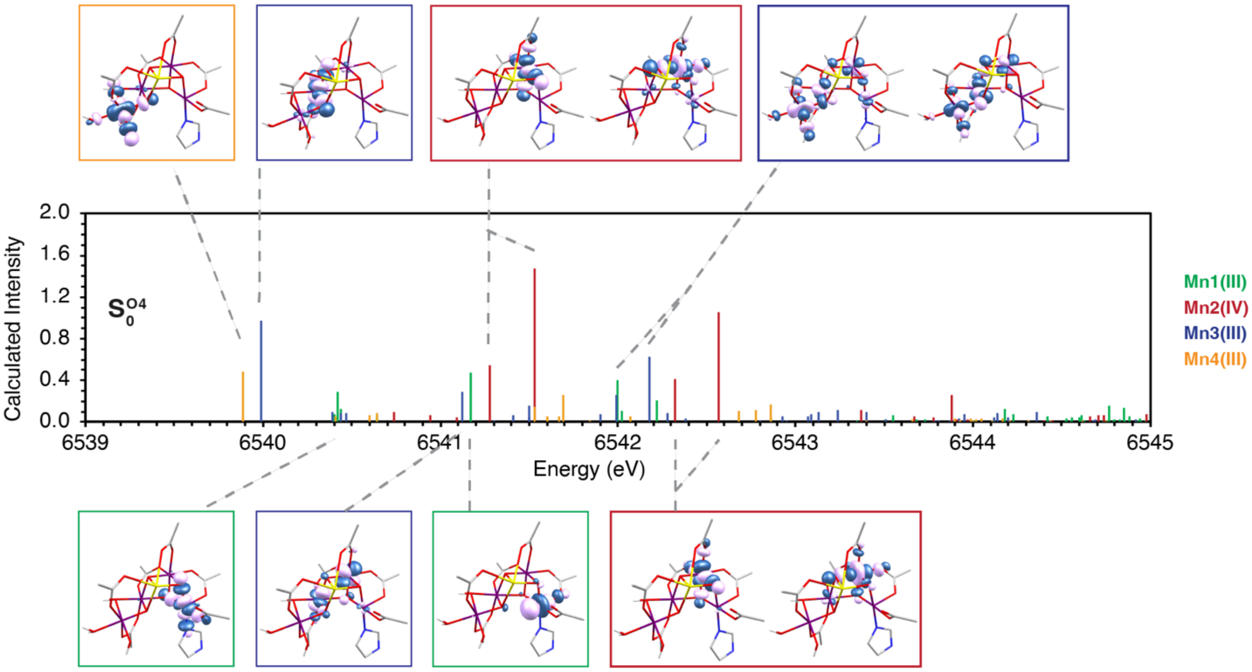

Figure S21. Assignment of the calculated pre-edge XAS spectrum based on the NTOs associated with the transitions for model $\mathbf{S_0^{O4}}$.

12. Analysis of Individual Mn Ion Contributions in the S_3 State Pre-edge XAS Spectra

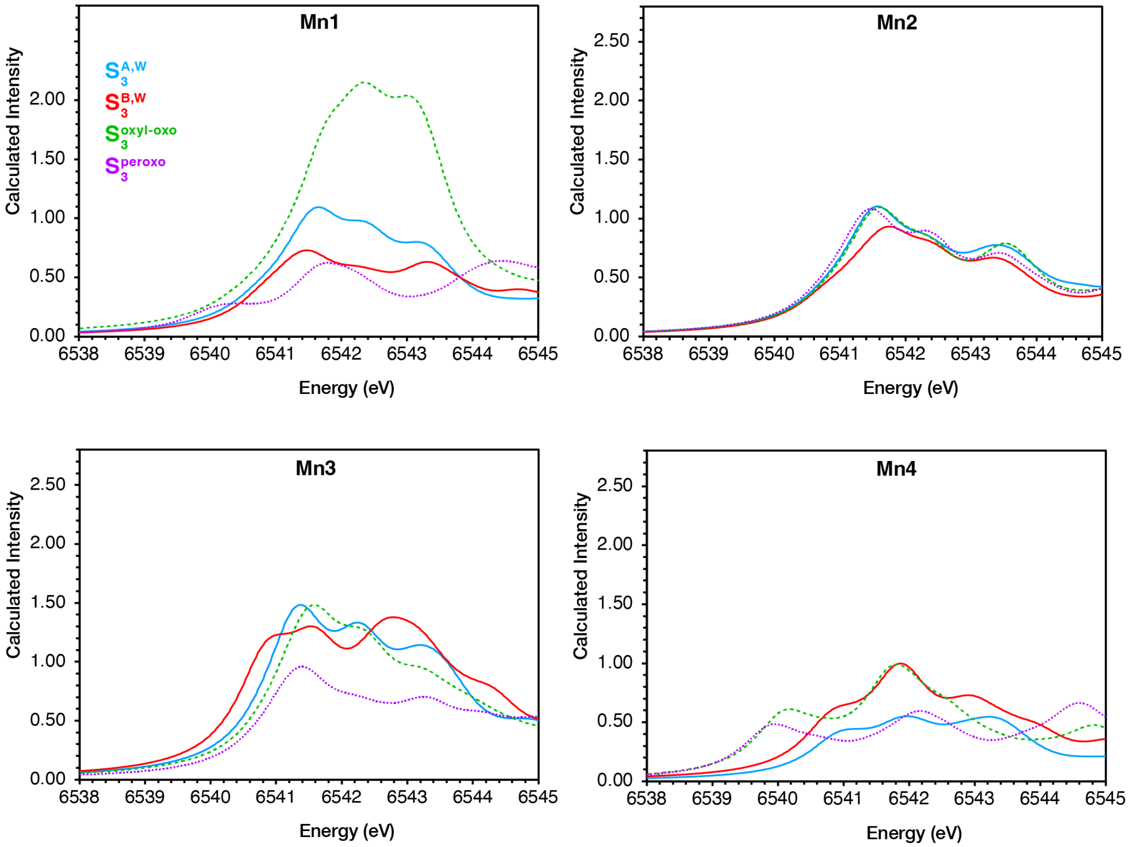

Figure S22. Calculated XAS spectra for individual Mn ions for $\mathbf{S_3^{A,W}}$, $\mathbf{S_3^{B,W}}$, $\mathbf{S_3^{oxyl-oxo}}$ and $\mathbf{S_3^{peroxo}}$. The higher intensity of the transitions of the calculated $\mathbf{S_3^{oxyl-oxo}}$ spectra compared to the $\mathbf{S_3^{A,W}}$ in the 6541–6544 eV region, is attributed mostly to excitations from Mn1 and Mn4 1s core orbitals, while the calculated $\mathbf{S_3^{peroxo}}$ spectra show lower overall intensity than $\mathbf{S_3^{A,W}}$ mostly due to excitations from Mn1 and Mn3 1s core orbitals.

13. Example of XAS Orca 5 Input

```
! UKS TPSSh RIJCOSX ZORA ZORA-def2-TZVP(-f) SARC/J CPCM
! NoTrah TightSCF

%pal nprocs 10 end
%maxcore 15000
%basis newgto C "ZORA-def2-SVP" end
       newgto H "ZORA-def2-SVP" end
end
%cpcm
    surfacetype vdw_gaussian
    epsilon 6.0
end
%scf maxiter 200
     shift shift 0.10 erroff 0.1 end
     FlipSpin 1,2 FinalMs 0.5
end
%tddft NRoots 150
        MaxDim 8
        OrbWin[0]=0,0,-1,-1
        OrbWin[1]=0,0,-1,-1
        DoQuad true
End

*xyzfile 1 14 filename.xyz
```

References

(1) Messinger, J.; Robblee, J. H.; Bergmann, U.; Fernandez, C.; Glatzel, P.; Visser, H.; Cinco, R. M.; McFarlane, K. L.; Bellacchio, E.; Pizarro, S. A.; Cramer, S. P.; Sauer, K.; Klein, M. P.; Yachandra, V. K. Absence of Mn-centered oxidation in the $S_2 \rightarrow S_3$ transition: implications for the mechanism of photosynthetic water oxidation. *J. Am. Chem. Soc.* **2001**, *123*, 7804-7820.

(2) Cox, N.; Retegan, M.; Neese, F.; Pantazis, D. A.; Boussac, A.; Lubitz, W. Electronic structure of the oxygen-evolving complex in photosystem II prior to O-O bond formation. *Science* **2014**, *345*, 804-808.

(3) Marchiori, D. A.; Debus, R. J.; Britt, R. D. Pulse EPR Spectroscopic Characterization of the S_3 State of the Oxygen-Evolving Complex of Photosystem II Isolated from *Synechocystis*. *Biochemistry* **2020**, *59*, 4864–4872.

(4) Chrysina, M.; Heyno, E.; Kutin, Y.; Reus, M.; Nilsson, H.; Nowaczyk, M. M.; DeBeer, S.; Neese, F.; Messinger, J.; Lubitz, W.; Cox, N. Five-coordinate Mn^{IV} intermediate in the activation of nature's water splitting cofactor. *Proc. Natl. Acad. Sci. U. S. A.* **2019**, *116*, 16841-16846.

(5) Zahariou, G.; Ioannidis, N.; Sanakis, Y.; Pantazis, D. A. Arrested Substrate Binding Resolves Catalytic Intermediates in Higher-Plant Water Oxidation. *Angew. Chem., Int. Ed.* **2021**, *60*, 3156-3162.

(6) Retegan, M.; Krewald, V.; Mamedov, F.; Neese, F.; Lubitz, W.; Cox, N.; Pantazis, D. A. A five-coordinate Mn(IV) intermediate in biological water oxidation: spectroscopic signature and a pivot mechanism for water binding. *Chem. Sci.* **2016**, *7*, 72-84.

(7) Haumann, M.; Muller, C.; Liebisch, P.; Iuzzolino, L.; Dittmer, J.; Grabolle, M.; Neisius, T.; Meyer-Klaucke, W.; Dau, H. Structural and oxidation state changes of the photosystem II manganese complex in four transitions of the water oxidation cycle ($S_0 \rightarrow S_1$, $S_1 \rightarrow S_2$, $S_2 \rightarrow S_3$, and $S_{3,4} \rightarrow S_0$) characterized by X-ray absorption spectroscopy at 20 K and room temperature. *Biochemistry* **2005**, *44*, 1894-1908.
